# Supplementary material for: Social environment and brain structure in adolescent mental health: A cross-sectional structural equation modelling study using IMAGEN data
Source: PLoS One. 2023 Jan 5;18(1):e0280062. doi: 10.1371/journal.pone.0280062 (PMC9815590; doi:10.1371/journal.pone.0280062)
Supplement: S1 File — (DOCX) [file pone.0280062.s001.docx]

## Supporting information

**S1 Appendix. Information about local ethics research committee approval at each study site at IMAGEN.**

London, England: Psychiatry, Nursing and Midwifery Research Ethics Subcommittee, Waterloo Campus, King’s College London; Nottingham, England: University of Nottingham Medical School Ethics Committee; Mannheim, Germany: Medizinische Fakultaet Mannheim, Ruprecht Karl Universitaet Heidelberg and Ethik-Kommission II an der Fakultaet fuer Kliniksche Medizin Mannheim; Dresden, Germany: Ethikkommission der Medizinischen Fakultaet Carl Gustav Carus, TU Dresden Medizinische Fakultaet; Hamburg, Germany: Ethics Board, Hamburg Chamber of Physicians; Paris, France: CPP IDF VII (Comité de protection des personnes Ile de France), ID RCB: 2007-A00778-45 September 24, 2007; Dublin, Ireland: TCD School of Psychology REC; and Berlin, Germany: Ethics Committee of the Faculty of Psychology.

**S2 Appendix. Results from measurement invariance analysis**

Results from all measurement invariance tests for the latent variables of socioeconomic stress, family support, peer problems and emotional symptoms are presented in S1 Table.

**S1 Table.** **Measurement invariance models for socioeconomic stress, family support, peer problems and emotional symptoms between sex (N = 1950).**

|  |  | **Robust χ^2^  goodness of fit** | | |  |  |  | **Robust χ^2^  difference test** | | |
| --- | --- | --- | --- | --- | --- | --- | --- | --- | --- | --- |
| **Model** |  | χ^2^ | df | *p* | CFI | RMSEA |  | χ^2^ | df | *p* |
| **Socioeconomic Stress Model** |  |  |  |  |  |  |  |  |  |  |
| Configural/Threshold Invariance |  | 22.863 | 4 | <.001 | 0.972 | 0.070 |  | - | - | *-* |
| Metric Invariance |  | 27.209 | 7 | <.001 | 0.970 | 0.054 |  | 3.258 | 3 | 0.354 |
| Scalar Invariance |  | 33.909 | 10 | <.001 | 0.965 | 0.050 |  | 5.880 | 3 | 0.118 |
| Strict Invariance |  | 38.685 | 14 | <.001 | 0.964 | 0.043 |  | 4.483 | 4 | 0.344 |
| **Family Support Model** |  |  |  |  |  |  |  |  |  |  |
| Configural Model |  | 3.393 | 4 | 0.494 | 1.00 | <.001 |  | - | - | *-* |
| Threshold Invariance |  | 2.685 | 8 | 0.953 | 1.00 | <.001 |  | 0.728 | 4 | 0.948 |
| Metric Invariance |  | 4.960 | 11 | 0.933 | 1.00 | <.001 |  | 2.179 | 3 | 0.536 |
| Scalar Invariance |  | 7.703 | 14 | 0.904 | 1.00 | <.001 |  | 2.841 | 3 | 0.417 |
| Strict Invariance |  | 12.069 | 18 | 0.844 | 1.00 | <.001 |  | 4.166 | 4 | 0.384 |
| **Peer Problems Model** |  |  |  |  |  |  |  |  |  |  |
| Configural/Threshold Invariance |  | 28.577 | 10 | 0.001 | 0.977 | 0.044 |  | - | - | *-* |
| Metric Invariance |  | 29.052 | 14 | 0.010 | 0.981 | 0.033 |  | 1.897 | 4 | 0.755 |
| Scalar Invariance |  | 36.627 | 18 | 0.006 | 0.977 | 0.033 |  | 6.841 | 4 | 0.145 |
| Strict Invariance |  | 47.168 | 23 | 0.002 | 0.970 | 0.033 |  | 9.189 | 5 | 0.102 |
| **Emotional Symptoms Model** |  |  |  |  |  |  |  |  |  |  |
| Configural/Threshold Invariance |  | 23.171 | 10 | 0.010 | 0.990 | 0.037 |  | - | - | *-* |
| Metric Invariance |  | 34.691 | 14 | 0.002 | 0.984 | 0.039 |  | 9.248 | 4 | 0.055 |
| Scalar Invariance |  | 43.438 | 18 | 0.001 | 0.981 | 0.038 |  | 8.106 | 4 | 0.088 |
| Strict Invariance |  | 47.728 | 23 | 0.002 | 0.981 | 0.033 |  | 4.287 | 5 | 0.509 |

First, the configural/threshold invariance model for socioeconomic stress was an adequate fit to the data, although the RMSEA was larger than the cut-off of 0.05. Subsequent models did not significantly differ in fit from the previous models – in fact, the RMSEA value improved with added equality constraints – therefore metric, scalar and strict invariance was achieved. This also allowed comparison of means between sex: there was no difference in parent-reported socioeconomic stress between parents of males compared to females (female to male estimate = 0.040, SE = 0.075, p = 0.595). In the strict invariance model, it was noted that the ‘problems with neighbours/the neighbourhood’ item had a weak loading for both males (β = 0.292) and females (0.320). Therefore, a separate model was tested which fixed these loadings to 0. This resulted in significantly worse model fit (Δχ2 = 28.561, Δdf = 1, p < .001), therefore this item was retained in the model.

For each of the socioeconomic stress indicators, non-zero data points ranged between 5.1% (problems with neighbours/neighbourhood - females) to 33.56% (financial difficulties - females). There appeared to be a floor effect due to the nature of the items – for example, many families may not have had problems with their neighbours or neighbourhood. Nonetheless, the latent variable of socioeconomic stress had a good fit and measurement invariance was achieved (see S1 Table). It was found that the ‘problems with neighbours/neighbourhood’ item had a low loading, which may have been due to the low non-zero options. However, it was retained in the model as removal of it resulted in worse fit. In terms of whether the model was affected by indicator items that were skewed towards zero values, the WLSMV estimator and robust fit statistics were used to address this.

The configural model for family support was an excellent fit to the data. Thus, the analysis proceeded by applying parameter constraints in successive models. Threshold, metric, scalar and strict invariance were achieved, showing that the item loadings, intercepts and residuals were equal between sexes. This also allowed comparison of means between sex: this revealed that there was no difference in parent-reported family support between sex (female to male estimate = -0.083, SE = 0.066, p = 0.205).

The configural/threshold invariance model for peer problems was a good fit to the data. Threshold, metric, scalar and strict invariance were achieved, showing that the item loadings, intercepts and residuals were equal between sexes. In terms of mean difference between sex, males had significantly greater peer problems than females (female to male estimate = 0.136, SE = 0.065, p = 0.036).

The configural/threshold invariance model for emotional symptoms was a good fit to the data. Threshold, metric, scalar and strict invariance were achieved, showing that the item loadings, intercepts and residuals were equal between sexes. In terms of mean difference between sex, males had significantly lower emotional symptoms mean score than females (female to male estimate = -0.926, SE = 0.075, p < 0.001). It was noted that the ‘somatic’ item had a weak loading for both males (β = 0.444) and females (0.422). Therefore, a separate model was tested which fixed these loadings to 0. This resulted in significantly worse model fit (Δχ2 = 216.89, Δdf = 1, p < .001), therefore this item was retained in the model.

Item loadings for the measurement invariance CFA models are presented in S2 Table.

**S2 Table.** **Item loadings for the individual strict invariance confirmatory factor analysis models, including estimate, standard error, standardised beta and p-value (N = 1950).**

|  | | **Males (n = 949)** | | | |  | **Females (n = 1001)** | | | |
| --- | --- | --- | --- | --- | --- | --- | --- | --- | --- | --- |
| **Latent Variable** | **Indicators** | **Estimate** | **SE** | **β** | **p-value** |  | **Estimate** | **SE** | **β** | **p-value** |
| Socioeconomic Stress | Unemployment | 1.080 | 0.161 | 0.698 | <0.001 |  | 1.080 | 0.161 | 0.734 | <0.001 |
|  | Financial | 1.968 | 0.576 | 0.872 | 0.001 |  | 1.968 | 0.576 | 0.892 | 0.001 |
|  | Home Inadequacy | 0.609 | 0.074 | 0.482 | <0.001 |  | 0.609 | 0.074 | 0.520 | <0.001 |
|  | Neighbourhood | 0.338 | 0.070 | 0.292 | <0.001 |  | 0.338 | 0.070 | 0.320 | <0.001 |
| Family Support | Praised and rewarded | 1.066 | 0.072 | 0.741 | <0.001 |  | 1.066 | 0.072 | 0.729 | <0.001 |
|  | Gets love and affection | 1.339 | 0.107 | 0.811 | <0.001 |  | 1.339 | 0.107 | 0.801 | <0.001 |
|  | Gets help and support | 1.018 | 0.074 | 0.725 | <0.001 |  | 1.018 | 0.074 | 0.714 | <0.001 |
|  | Liked and respected | 0.959 | 0.074 | 0.705 | <0.001 |  | 0.959 | 0.074 | 0.692 | <0.001 |
| Peer Problems | Loner | 0.619 | 0.056 | 0.546 | <0.001 |  | 0.619 | 0.056 | 0.527 | <0.001 |
|  | Friend | -0.597 | 0.078 | -0.532 | <0.001 |  | -0.597 | 0.078 | -0.513 | <0.001 |
|  | Popular | -0.699 | 0.056 | -0.592 | <0.001 |  | -0.699 | 0.056 | -0.573 | <0.001 |
|  | Bullied | 0.911 | 0.099 | 0.691 | <0.001 |  | 0.911 | 0.099 | 0.673 | <0.001 |
|  | Old Best | 0.613 | 0.053 | 0.541 | <0.001 |  | 0.613 | 0.053 | 0.523 | <0.001 |
| Emotional Symptoms | Unhappy | 0.987 | 0.076 | 0.724 | <0.001 |  | 0.987 | 0.076 | 0.703 | <0.001 |
|  | Worries | 0.879 | 0.059 | 0.683 | <0.001 |  | 0.879 | 0.059 | 0.660 | <0.001 |
|  | Somatic | 0.465 | 0.040 | 0.444 | <0.001 |  | 0.465 | 0.040 | 0.422 | <0.001 |
|  | Clingy | 0.560 | 0.042 | 0.512 | <0.001 |  | 0.560 | 0.042 | 0.489 | <0.001 |
|  | Afraid | 0.727 | 0.054 | 0.612 | <0.001 |  | 0.727 | 0.054 | 0.588 | <0.001 |

**S3 Table. Regression statistics for model 1, nested within model 2 with WBV paths fixed to 0.**

|  |  |  |  | | **Males (n = 949)** | | | |  | **Females (n = 1001)** | | | | | |
| --- | --- | --- | --- | --- | --- | --- | --- | --- | --- | --- | --- | --- | --- | --- | --- |
| **Outcome** | **Predictor** | **Estimate** | **CI lower** | **CI upper** | | **SE** | **β** | **p-value** |  | **Estimate** | **CI lower** | **CI upper** | **SE** | **β** | **p-value** |
| Emotional Symptoms | Psychiatric Diagnosis (Yes) | 0.213 | -0.117 | 0.544 | | 0.169 | 0.052 | 0.206 |  | **0.743** | **0.460** | **1.026** | **0.144** | **0.210** | **<0.001** |
|  | Recruitment Centre (Dresden) | -0.322 | -0.746 | 0.102 | | 0.216 | -0.084 | 0.137 |  | 0.303 | -0.087 | 0.693 | 0.199 | 0.078 | 0.128 |
|  | Recruitment Centre (Dublin) | 0.320 | -0.171 | 0.811 | | 0.250 | 0.074 | 0.201 |  | **0.498** | **0.087** | **0.908** | **0.209** | **0.111** | **0.017** |
|  | Recruitment Centre (Hamburg) | -0.290 | -0.698 | 0.118 | | 0.208 | -0.072 | 0.164 |  | 0.227 | -0.139 | 0.593 | 0.187 | 0.061 | 0.224 |
|  | Recruitment Centre (London) | **0.618** | **0.196** | **1.040** | | **0.215** | **0.153** | **0.004** |  | 0.252 | -0.118 | 0.621 | 0.189 | 0.067 | 0.182 |
|  | Recruitment Centre (Mannheim) | -0.443 | -0.909 | 0.024 | | 0.238 | -0.106 | 0.063 |  | -0.132 | -0.544 | 0.281 | 0.211 | -0.033 | 0.532 |
|  | Recruitment Centre (Nottingham) | 0.266 | -0.122 | 0.654 | | 0.198 | 0.079 | 0.179 |  | 0.003 | -0.352 | 0.357 | 0.181 | 0.001 | 0.988 |
|  | Recruitment Centre (Paris) | 0.312 | -0.135 | 0.759 | | 0.228 | 0.082 | 0.171 |  | 0.281 | -0.138 | 0.700 | 0.214 | 0.073 | 0.189 |
|  | Mean PDS score | -0.102 | -0.289 | 0.085 | | 0.096 | -0.042 | 0.286 |  | -0.045 | -0.273 | 0.183 | 0.116 | -0.015 | 0.697 |
|  | Family Support | 0.009 | -0.112 | 0.130 | | 0.062 | 0.008 | 0.889 |  | -0.119 | -0.246 | 0.007 | 0.064 | -0.104 | 0.064 |
|  | Peer Problems | **0.718** | **0.571** | **0.866** | | **0.075** | **0.622** | **<0.001** |  | **0.568** | **0.406** | **0.730** | **0.083** | **0.495** | **<0.001** |
|  | Socioeconomic Stress | **-0.159** | **-0.316** | **-0.003** | | **0.080** | **-0.115** | **0.046** |  | -0.010 | -0.153 | 0.133 | 0.073 | -0.008 | 0.889 |
|  | Whole Brain Volume | 0 | 0.000 | 0.000 | | - | 0 | - |  | 0 | 0 | 0 | - | 0 | - |
|  | Amygdala GMV | 0.133 | -0.117 | 0.383 | | 0.127 | 0.045 | 0.297 |  | -0.037 | -0.282 | 0.207 | 0.125 | -0.012 | 0.765 |
|  | vmPFC GMV | -0.068 | -0.144 | 0.007 | | 0.039 | -0.077 | 0.076 |  | 0.010 | -0.068 | 0.088 | 0.040 | 0.010 | 0.807 |
| Family Support | **Psychiatric Diagnosis (Yes)** | **-0.348** | **-0.646** | **-0.049** | | **0.152** | **-0.095** | **0.023** |  | **-0.475** | **-0.748** | **-0.203** | **0.139** | **-0.155** | **0.001** |
|  | Recruitment Centre (Dresden) | 0.093 | -0.283 | 0.469 | | 0.192 | 0.027 | 0.628 |  | -0.037 | -0.423 | 0.350 | 0.197 | -0.011 | 0.852 |
|  | Recruitment Centre (Dublin) | -0.086 | -0.472 | 0.300 | | 0.197 | -0.022 | 0.662 |  | -0.102 | -0.498 | 0.295 | 0.202 | -0.026 | 0.615 |
|  | Recruitment Centre (Hamburg) | **0.420** | **0.035** | **0.806** | | **0.197** | **0.118** | **0.033** |  | 0.057 | -0.290 | 0.404 | 0.177 | 0.018 | 0.747 |
|  | Recruitment Centre (London) | -0.022 | -0.404 | 0.361 | | 0.195 | -0.006 | 0.911 |  | -0.245 | -0.603 | 0.113 | 0.183 | -0.075 | 0.180 |
|  | Recruitment Centre (Mannheim) | 0.301 | -0.095 | 0.697 | | 0.202 | 0.081 | 0.136 |  | -0.186 | -0.559 | 0.188 | 0.191 | -0.054 | 0.330 |
|  | Recruitment Centre (Nottingham) | -0.276 | -0.615 | 0.062 | | 0.173 | -0.093 | 0.110 |  | **-0.453** | **-0.790** | **-0.116** | **0.172** | **-0.152** | **0.008** |
|  | Recruitment Centre (Paris) | **-0.418** | **-0.764** | **-0.072** | | **0.176** | **-0.124** | **0.018** |  | **-0.400** | **-0.777** | **-0.023** | **0.192** | **-0.120** | **0.038** |
|  | Mean PDS score | 0.097 | -0.079 | 0.274 | | 0.090 | 0.045 | 0.280 |  | 0.090 | -0.118 | 0.298 | 0.106 | 0.035 | 0.395 |
|  | Peer Problems | -0.081 | -0.196 | 0.034 | | 0.059 | -0.079 | 0.167 |  | 0.008 | -0.112 | 0.127 | 0.061 | 0.008 | 0.902 |
|  | Socioeconomic Stress | **-0.230** | **-0.356** | **-0.104** | | **0.064** | **-0.187** | **<0.001** |  | **-0.374** | **-0.505** | **-0.244** | **0.067** | **-0.342** | **<0.001** |
| Peer Problems | Psychiatric Diagnosis (Yes) | **0.785** | **0.489** | **1.081** | | **0.151** | **0.221** | **<0.001** |  | **0.923** | **0.659** | **1.186** | **0.134** | **0.299** | **<0.001** |
|  | Recruitment Centre (Dresden) | **0.456** | **0.079** | **0.833** | | **0.192** | **0.138** | **0.018** |  | **0.545** | **0.182** | **0.908** | **0.185** | **0.161** | **0.003** |
|  | Recruitment Centre (Dublin) | **-0.480** | **-0.868** | **-0.092** | | **0.198** | **-0.129** | **0.015** |  | -0.382 | -0.781 | 0.018 | 0.204 | -0.098 | 0.061 |
|  | Recruitment Centre (Hamburg) | 0.027 | -0.350 | 0.405 | | 0.193 | 0.008 | 0.887 |  | 0.066 | -0.299 | 0.432 | 0.186 | 0.021 | 0.722 |
|  | Recruitment Centre (London) | -0.209 | -0.578 | 0.160 | | 0.188 | -0.060 | 0.268 |  | 0.259 | -0.097 | 0.614 | 0.181 | 0.079 | 0.154 |
|  | Recruitment Centre (Mannheim) | 0.157 | -0.252 | 0.565 | | 0.208 | 0.043 | 0.452 |  | 0.228 | -0.134 | 0.591 | 0.185 | 0.066 | 0.217 |
|  | Recruitment Centre (Nottingham) | 0.048 | -0.298 | 0.395 | | 0.177 | 0.017 | 0.784 |  | -0.038 | -0.379 | 0.303 | 0.174 | -0.013 | 0.827 |
|  | Recruitment Centre (Paris) | **-0.598** | **-0.993** | **-0.203** | | **0.202** | **-0.182** | **0.003** |  | **-0.588** | **-0.975** | **-0.200** | **0.198** | **-0.175** | **0.003** |
|  | Mean PDS score | -0.065 | -0.242 | 0.112 | | 0.090 | -0.031 | 0.474 |  | 0.071 | -0.149 | 0.292 | 0.113 | 0.028 | 0.526 |
|  | Socioeconomic Stress | 0.072 | -0.069 | 0.214 | | 0.072 | 0.060 | 0.318 |  | 0.099 | -0.017 | 0.216 | 0.060 | 0.090 | 0.095 |
| Whole Brain Volume | Psychiatric Diagnosis (Yes) | 0 | 0 | 0 | | - | 0 | - |  | 0 | 0 | 0 | - | 0 | - |
|  | Recruitment Centre (Dresden) | 0 | 0 | 0 | | - | 0 | - |  | 0 | 0 | 0 | - | 0 | - |
|  | Recruitment Centre (Dublin) | 0 | 0 | 0 | | - | 0 | - |  | 0 | 0 | 0 | - | 0 | - |
|  | Recruitment Centre (Hamburg) | 0 | 0 | 0 | | - | 0 | - |  | 0 | 0 | 0 | - | 0 | - |
|  | Recruitment Centre (London) | 0 | 0 | 0 | | - | 0 | - |  | 0 | 0 | 0 | - | 0 | - |
|  | Recruitment Centre (Mannheim) | 0 | 0 | 0 | | - | 0 | - |  | 0 | 0 | 0 | - | 0 | - |
|  | Recruitment Centre (Nottingham) | 0 | 0 | 0 | | - | 0 | - |  | 0 | 0 | 0 | - | 0 | - |
|  | Recruitment Centre (Paris) | 0 | 0 | 0 | | - | 0 | - |  | 0 | 0 | 0 | - | 0 | - |
|  | Mean PDS score | 0 | 0 | 0 | | - | 0 | - |  | 0 | 0 | 0 | - | 0 | - |
|  | Family Support | 0 | 0 | 0 | | - | 0 | - |  | 0 | 0 | 0 | - | 0 | - |
|  | Peer Problems | 0 | 0 | 0 | | - | 0 | - |  | 0 | 0 | 0 | - | 0 | - |
|  | Socioeconomic Stress | 0 | 0 | 0 | | - | 0 | - |  | 0 | 0 | 0 | - | 0 | - |
| Amygdala GMV | Psychiatric Diagnosis (Yes) | -0.019 | -0.111 | 0.073 | | 0.047 | -0.014 | 0.688 |  | **-0.088** | **-0.166** | **-0.009** | **0.040** | **-0.075** | **0.029** |
|  | Recruitment Centre (Dresden) | -0.025 | -0.132 | 0.082 | | 0.055 | -0.019 | 0.651 |  | 0.040 | -0.058 | 0.139 | 0.050 | 0.032 | 0.424 |
|  | Recruitment Centre (Dublin) | 0.062 | -0.064 | 0.187 | | 0.064 | 0.042 | 0.335 |  | 0.065 | -0.038 | 0.167 | 0.052 | 0.044 | 0.214 |
|  | Recruitment Centre (Hamburg) | 0.059 | -0.051 | 0.170 | | 0.056 | 0.044 | 0.293 |  | -0.016 | -0.113 | 0.081 | 0.049 | -0.013 | 0.743 |
|  | Recruitment Centre (London) | -0.085 | -0.201 | 0.031 | | 0.059 | -0.062 | 0.151 |  | **-0.251** | **-0.348** | **-0.154** | **0.049** | **-0.204** | **<0.001** |
|  | Recruitment Centre (Mannheim) | -0.008 | -0.111 | 0.095 | | 0.052 | -0.006 | 0.881 |  | **-0.104** | **-0.201** | **-0.008** | **0.049** | **-0.080** | **0.035** |
|  | Recruitment Centre (Nottingham) | **0.222** | **0.121** | **0.322** | | **0.051** | **0.195** | **<0.001** |  | **0.225** | **0.133** | **0.317** | **0.047** | **0.200** | **<0.001** |
|  | Recruitment Centre (Paris) | 0.085 | -0.026 | 0.196 | | 0.057 | 0.066 | 0.133 |  | 0.047 | -0.055 | 0.148 | 0.052 | 0.037 | 0.367 |
|  | Mean PDS score | **0.063** | **0.009** | **0.116** | | **0.027** | **0.076** | **0.021** |  | **0.067** | **0.005** | **0.129** | **0.032** | **0.069** | **0.035** |
|  | Whole Brain Volume | 0 | 0 | 0 | | - | 0 | - |  | 0 | 0 | 0 | - | 0 | - |
|  | Family Support | 0.002 | -0.028 | 0.032 | | 0.015 | 0.005 | 0.900 |  | 0.007 | -0.026 | 0.040 | 0.017 | 0.018 | 0.682 |
|  | Peer Problems | -0.007 | -0.045 | 0.031 | | 0.019 | -0.018 | 0.718 |  | 0.014 | -0.020 | 0.049 | 0.018 | 0.038 | 0.411 |
|  | Socioeconomic Stress | **-0.046** | **-0.088** | **-0.004** | | **0.022** | **-0.098** | **0.033** |  | -0.032 | -0.070 | 0.007 | 0.020 | -0.076 | 0.109 |
| vmPFC GMV | Psychiatric Diagnosis (Yes) | -0.085 | -0.376 | 0.206 | | 0.148 | -0.018 | 0.568 |  | -0.090 | -0.330 | 0.150 | 0.123 | -0.025 | 0.463 |
|  | Recruitment Centre (Dresden) | **-0.647** | **-1.019** | **-0.274** | | **0.190** | **-0.150** | **0.001** |  | -0.120 | -0.445 | 0.205 | 0.166 | -0.030 | 0.469 |
|  | Recruitment Centre (Dublin) | **0.826** | **0.430** | **1.222** | | **0.202** | **0.170** | **<0.001** |  | **1.245** | **0.914** | **1.575** | **0.169** | **0.270** | **<0.001** |
|  | Recruitment Centre (Hamburg) | -0.100 | -0.494 | 0.293 | | 0.201 | -0.022 | 0.617 |  | -0.154 | -0.458 | 0.150 | 0.155 | -0.040 | 0.320 |
|  | Recruitment Centre (London) | 0.323 | -0.050 | 0.696 | | 0.190 | 0.071 | 0.089 |  | 0.189 | -0.095 | 0.473 | 0.145 | 0.049 | 0.193 |
|  | Recruitment Centre (Mannheim) | **-0.673** | **-1.041** | **-0.304** | | **0.188** | **-0.142** | **<0.001** |  | **-0.496** | **-0.810** | **-0.182** | **0.160** | **-0.121** | **0.002** |
|  | Recruitment Centre (Nottingham) | **0.482** | **0.135** | **0.830** | | **0.177** | **0.128** | **0.007** |  | **0.531** | **0.233** | **0.828** | **0.152** | **0.150** | **<0.001** |
|  | Recruitment Centre (Paris) | 0.165 | -0.254 | 0.584 | | 0.214 | 0.039 | 0.441 |  | 0.119 | -0.209 | 0.447 | 0.167 | 0.030 | 0.477 |
|  | Mean PDS score | 0.052 | -0.113 | 0.217 | | 0.084 | 0.019 | 0.538 |  | 0.039 | -0.135 | 0.213 | 0.089 | 0.013 | 0.661 |
|  | Whole Brain Volume | 0 | 0 | 0 | | - | 0 | - |  | 0 | 0 | 0 | - | 0 | - |
|  | Family Support | 0.096 | -0.005 | 0.196 | | 0.051 | 0.076 | 0.063 |  | 0.015 | -0.092 | 0.122 | 0.055 | 0.012 | 0.787 |
|  | Peer Problems | 0.024 | -0.093 | 0.141 | | 0.060 | 0.019 | 0.683 |  | -0.053 | -0.156 | 0.050 | 0.053 | -0.045 | 0.313 |
|  | Socioeconomic Stress | -0.080 | -0.222 | 0.063 | | 0.073 | -0.051 | 0.274 |  | **-0.160** | **-0.278** | **-0.043** | **0.060** | **-0.124** | **0.008** |
| Covariance | Amygdala and vmPFC | **0.176** | **0.138** | **0.213** | | **0.019** | **0.303** | **<0.001** |  | **0.155** | **0.126** | **0.185** | **0.015** | **0.333** | **<0.001** |

Note: Statistically significant values (p < .05) are in bold for ease of reading. For recruitment centre, the reference category is Berlin. WBV values were divided by 1,000,000. Amygdala and vmPFC GMV values were divided by 1,000.

**S4 Table. Regression statistics for model 2, with WBV paths freely estimated.**

|  |  | **Males (n = 949)** | | | | | |  | **Females (n = 1001)** | | | | | |
| --- | --- | --- | --- | --- | --- | --- | --- | --- | --- | --- | --- | --- | --- | --- |
| **Outcome** | **Predictor** | **Estimate** | **CI lower** | **CI upper** | **SE** | **β** | **p-value** |  | **Estimate** | **CI lower** | **CI upper** | **SE** | **β** | **p-value** |
| Emotional Symptoms | Psychiatric Diagnosis (Yes) | 0.237 | -0.095 | 0.569 | 0.169 | 0.058 | 0.161 |  | **0.743** | 0.460 | 1.026 | **0.136** | **0.210** | **<0.001** |
|  | Recruitment Centre (Dresden) | -0.305 | -0.731 | 0.121 | 0.217 | -0.080 | 0.161 |  | 0.304 | -0.086 | 0.693 | 0.196 | 0.078 | 0.127 |
|  | Recruitment Centre (Dublin) | 0.306 | -0.190 | 0.802 | 0.253 | 0.071 | 0.226 |  | **0.499** | **0.088** | **0.909** | **0.206** | **0.111** | **0.017** |
|  | Recruitment Centre (Hamburg) | -0.253 | -0.660 | 0.153 | 0.207 | -0.063 | 0.222 |  | 0.227 | -0.140 | 0.593 | 0.184 | 0.061 | 0.225 |
|  | Recruitment Centre (London) | **0.597** | **0.170** | **1.023** | **0.218** | **0.148** | **0.006** |  | 0.254 | -0.116 | 0.625 | 0.184 | 0.068 | 0.178 |
|  | Recruitment Centre (Mannheim) | -0.440 | -0.907 | 0.027 | 0.238 | -0.105 | 0.065 |  | -0.131 | -0.543 | 0.282 | 0.202 | -0.033 | 0.535 |
|  | Recruitment Centre (Nottingham) | 0.301 | -0.091 | 0.693 | 0.200 | 0.090 | 0.133 |  | -0.001 | -0.356 | 0.356 | 0.179 | -0.001 | 0.999 |
|  | Recruitment Centre (Paris) | 0.300 | -0.149 | 0.748 | 0.229 | 0.079 | 0.190 |  | 0.281 | -0.137 | 0.700 | 0.207 | 0.073 | 0.188 |
|  | Mean PDS score | -0.114 | -0.301 | 0.074 | 0.096 | -0.047 | 0.235 |  | -0.045 | -0.273 | 0.183 | 0.113 | -0.015 | 0.700 |
|  | Family Support | 0.007 | -0.114 | 0.128 | 0.062 | 0.006 | 0.908 |  | -0.119 | -0.246 | 0.007 | 0.063 | -0.104 | 0.065 |
|  | Peer Problems | **0.717** | **0.570** | **0.865** | **0.075** | **0.621** | **<0.001** |  | **0.568** | **0.405** | **0.730** | **0.080** | **0.495** | **<0.001** |
|  | Socioeconomic Stress | **-0.159** | **-0.316** | **-0.003** | **0.080** | **-0.115** | **0.046** |  | -0.011 | -0.156 | 0.133 | 0.072 | -0.009 | 0.877 |
|  | Whole Brain Volume | 1.194 | -0.334 | 2.723 | 0.780 | 0.099 | 0.126 |  | -0.106 | -1.621 | 1.408 | 0.754 | -0.008 | 0.890 |
|  | Amygdala GMV | 0.060 | -0.210 | 0.329 | 0.137 | 0.020 | 0.664 |  | -0.029 | -0.298 | 0.240 | 0.134 | -0.009 | 0.835 |
|  | vmPFC GMV | **-0.122** | **-0.226** | **-0.019** | **0.053** | **-0.138** | **0.021** |  | 0.014 | -0.085 | 0.113 | 0.050 | 0.014 | 0.784 |
| Family Support | **Psychiatric Diagnosis (Yes)** | **-0.348** | **-0.647** | **-0.049** | **0.153** | **-0.095** | **0.023** |  | **-0.476** | **-0.749** | **-0.204** | **0.132** | **-0.155** | **0.001** |
|  | Recruitment Centre (Dresden) | 0.093 | -0.283 | 0.470 | 0.192 | 0.027 | 0.627 |  | -0.037 | -0.424 | 0.350 | 0.197 | -0.011 | 0.815 |
|  | Recruitment Centre (Dublin) | -0.086 | -0.472 | 0.300 | 0.197 | -0.022 | 0.662 |  | -0.102 | -0.499 | 0.295 | 0.203 | -0.026 | 0.615 |
|  | Recruitment Centre (Hamburg) | **0.421** | **0.035** | **0.807** | **0.197** | **0.118** | **0.033** |  | 0.057 | -0.290 | 0.404 | 0.177 | 0.018 | 0.747 |
|  | Recruitment Centre (London) | -0.022 | -0.405 | 0.361 | 0.195 | -0.006 | 0.911 |  | -0.246 | -0.604 | 0.113 | 0.182 | -0.075 | 0.179 |
|  | Recruitment Centre (Mannheim) | 0.301 | -0.095 | 0.698 | 0.202 | 0.081 | 0.136 |  | -0.186 | -0.560 | 0.188 | 0.184 | -0.054 | 0.329 |
|  | Recruitment Centre (Nottingham) | -0.277 | -0.616 | 0.062 | 0.173 | -0.093 | 0.110 |  | **-0.454** | **-0.791** | **-0.116** | **0.173** | **-0.152** | **0.008** |
|  | Recruitment Centre (Paris) | **-0.418** | **-0.764** | **-0.072** | **0.177** | **-0.124** | **0.018** |  | **-0.400** | **-0.777** | **-0.022** | **0.193** | **-0.120** | **0.038** |
|  | Mean PDS score | 0.097 | -0.079 | 0.274 | 0.090 | 0.045 | 0.280 |  | 0.090 | -0.118 | 0.298 | 0.106 | 0.035 | 0.395 |
|  | Peer Problems | -0.081 | -0.197 | 0.034 | 0.059 | -0.079 | 0.167 |  | 0.008 | -0.112 | 0.127 | 0.060 | 0.008 | 0.897 |
|  | Socioeconomic Stress | **-0.230** | **-0.356** | **-0.104** | **0.064** | **-0.187** | **<0.001** |  | **-0.376** | **-0.507** | **-0.245** | **0.067** | **-0.343** | **<0.001** |
| Peer Problems | Psychiatric Diagnosis (Yes) | **0.785** | **0.489** | **1.082** | **0.151** | **0.221** | **<0.001** |  | **0.923** | **0.659** | **1.186** | **0.125** | **0.299** | **<0.001** |
|  | Recruitment Centre (Dresden) | **0.456** | **0.079** | **0.833** | **0.192** | **0.138** | **0.018** |  | **0.545** | **0.182** | **0.908** | **0.183** | **0.161** | **0.003** |
|  | Recruitment Centre (Dublin) | **-0.480** | **-0.868** | **-0.091** | **0.198** | **-0.129** | **0.015** |  | -0.382 | -0.781 | 0.018 | 0.202 | -0.098 | 0.061 |
|  | Recruitment Centre (Hamburg) | 0.027 | -0.350 | 0.405 | 0.193 | -0.008 | 0.887 |  | 0.066 | -0.299 | 0.432 | 0.183 | 0.020 | 0.723 |
|  | Recruitment Centre (London) | -0.208 | -0.578 | 0.161 | 0.188 | -0.060 | 0.268 |  | 0.259 | -0.097 | 0.614 | 0.180 | 0.079 | 0.153 |
|  | Recruitment Centre (Mannheim) | 0.157 | -0.251 | 0.565 | 0.208 | 0.043 | 0.452 |  | 0.229 | -0.134 | 0.591 | 0.179 | 0.066 | 0.217 |
|  | Recruitment Centre (Nottingham) | 0.049 | -0.298 | 0.395 | 0.177 | 0.017 | 0.783 |  | -0.038 | -0.379 | 0.303 | 0.172 | -0.013 | 0.828 |
|  | Recruitment Centre (Paris) | **-0.598** | **-0.993** | **-0.203** | **0.202** | **-0.182** | **0.003** |  | **-0.588** | **-0.975** | **-0.200** | **0.194** | **-0.175** | **0.003** |
|  | Mean PDS score | -0.065 | -0.242 | 0.112 | 0.090 | -0.031 | 0.474 |  | 0.071 | -0.149 | 0.292 | 0.111 | 0.028 | 0.526 |
|  | Socioeconomic Stress | 0.072 | -0.070 | 0.214 | 0.072 | 0.060 | 0.320 |  | 0.100 | -0.017 | 0.217 | 0.059 | 0.091 | 0.095 |
| Whole Brain Volume | Psychiatric Diagnosis (Yes) | **-0.025** | **-0.046** | **-0.004** | **0.011** | **-0.073** | **0.020** |  | -0.006 | -0.024 | 0.012 | 0.008 | -0.021 | 0.540 |
|  | Recruitment Centre (Dresden) | **-0.045** | **-0.073** | **-0.018** | **0.014** | **-0.142** | **0.001** |  | 0.001 | -0.023 | 0.025 | 0.012 | 0.003 | 0.935 |
|  | Recruitment Centre (Dublin) | **0.052** | **0.020** | **0.085** | **0.017** | **0.146** | **0.002** |  | **0.069** | **0.044** | **0.093** | **0.013** | **0.207** | **<0.001** |
|  | Recruitment Centre (Hamburg) | **-0.031** | **-0.060** | **-0.003** | **0.015** | **-0.094** | **0.032** |  | -0.013 | -0.036 | 0.010 | 0.012 | -0.047 | 0.262 |
|  | Recruitment Centre (London) | 0.027 | -0.001 | 0.056 | 0.015 | 0.081 | 0.063 |  | 0.014 | -0.009 | 0.036 | 0.012 | 0.049 | 0.237 |
|  | Recruitment Centre (Mannheim) | **-0.033** | **-0.060** | **-0.006** | **0.014** | **-0.095** | **0.018** |  | -0.019 | -0.042 | 0.004 | 0.011 | -0.066 | 0.099 |
|  | Recruitment Centre (Nottingham) | 0.006 | -0.021 | 0.034 | 0.014 | 0.023 | 0.643 |  | 0.019 | -0.004 | 0.041 | 0.011 | 0.074 | 0.100 |
|  | Recruitment Centre (Paris) | 0.023 | -0.007 | 0.053 | 0.015 | 0.072 | 0.138 |  | 0.015 | -0.009 | 0.040 | 0.012 | 0.054 | 0.214 |
|  | Mean PDS score | **0.016** | **0.004** | **0.028** | **0.006** | **0.079** | **0.011** |  | 0.011 | -0.003 | 0.026 | 0.007 | 0.051 | 0.126 |
|  | Family Support | 0.006 | -0.002 | 0.013 | 0.004 | 0.060 | 0.138 |  | 0.003 | -0.005 | 0.010 | 0.004 | 0.034 | 0.445 |
|  | Peer Problems | 0.002 | -0.007 | 0.010 | 0.004 | 0.016 | 0.719 |  | -0.003 | -0.011 | 0.005 | 0.004 | -0.038 | 0.418 |
|  | Socioeconomic Stress | -0.007 | -0.017 | 0.003 | 0.005 | -0.059 | 0.177 |  | **-0.012** | **-0.021** | **-0.003** | **0.004** | **-0.127** | **0.007** |
| Amygdala GMV | Psychiatric Diagnosis (Yes) | 0.026 | -0.057 | 0.108 | 0.042 | 0.018 | 0.540 |  | **-0.075** | **-0.143** | **-0.007** | **0.032** | **-0.064** | **0.032** |
|  | Recruitment Centre (Dresden) | 0.057 | -0.046 | 0.160 | 0.053 | 0.044 | 0.280 |  | 0.038 | -0.053 | 0.129 | 0.046 | 0.030 | 0.415 |
|  | Recruitment Centre (Dublin) | -0.033 | -0.148 | 0.083 | 0.059 | -0.022 | 0.581 |  | **-0.094** | **-0.186** | **-0.003** | **0.046** | **-0.064** | **0.042** |
|  | Recruitment Centre (Hamburg) | **0.116** | **0.007** | **0.225** | **0.056** | **0.085** | **0.037** |  | 0.014 | -0.074 | 0.102 | 0.044 | 0.012 | 0.754 |
|  | Recruitment Centre (London) | **-0.134** | **-0.241** | **-0.027** | **0.054** | **-0.098** | **0.014** |  | **-0.283** | **-0.372** | **-0.194** | **0.045** | **-0.230** | **<0.001** |
|  | Recruitment Centre (Mannheim) | 0.052 | -0.043 | 0.146 | 0.048 | 0.036 | 0.283 |  | -0.059 | -0.144 | 0.026 | 0.042 | -0.046 | 0.171 |
|  | Recruitment Centre (Nottingham) | **0.210** | **0.119** | **0.300** | **0.046** | **0.184** | **<0.001** |  | **0.182** | **0.102** | **0.262** | **0.041** | **0.161** | **<0.001** |
|  | Recruitment Centre (Paris) | 0.044 | -0.057 | 0.145 | 0.052 | 0.034 | 0.394 |  | 0.011 | -0.076 | 0.098 | 0.044 | 0.009 | 0.808 |
|  | Mean PDS score | 0.034 | -0.016 | 0.084 | 0.025 | 0.041 | 0.179 |  | 0.041 | -0.013 | 0.094 | 0.027 | 0.042 | 0.135 |
|  | Whole Brain Volume | **1.804** | **1.575** | **2.032** | **0.116** | **0.442** | **<0.001** |  | **2.325** | **2.108** | **2.542** | **0.108** | **0.633** | **<0.001** |
|  | Family Support | -0.008 | -0.036 | 0.019 | 0.014 | -0.021 | 0.558 |  | 0.001 | -0.027 | 0.027 | 0.013 | 0.001 | 0.994 |
|  | Peer Problems | -0.010 | -0.043 | 0.024 | 0.017 | -0.025 | 0.566 |  | 0.022 | -0.007 | 0.051 | 0.015 | 0.058 | 0.140 |
|  | Socioeconomic Stress | -0.034 | -0.072 | 0.005 | 0.020 | -0.072 | 0.086 |  | -0.004 | -0.037 | 0.028 | 0.016 | -0.010 | 0.803 |
| vmPFC GMV | Psychiatric Diagnosis (Yes) | 0.142 | -0.077 | 0.361 | 0.112 | 0.031 | 0.205 |  | -0.041 | -0.225 | 0.142 | 0.086 | -0.011 | 0.661 |
|  | Recruitment Centre (Dresden) | -0.233 | -0.520 | 0.053 | 0.146 | -0.054 | 0.111 |  | -0.129 | -0.379 | 0.121 | 0.126 | -0.032 | 0.331 |
|  | Recruitment Centre (Dublin) | **0.348** | **0.042** | **0.654** | **0.156** | **0.072** | **0.026** |  | **0.643** | **0.402** | **0.883** | **0.122** | **0.140** | **<0.001** |
|  | Recruitment Centre (Hamburg) | 0.187 | -0.109 | 0.484 | 0.151 | 0.042 | 0.215 |  | -0.040 | -0.271 | 0.191 | 0.117 | -0.010 | 0.736 |
|  | Recruitment Centre (London) | 0.075 | -0.216 | 0.366 | 0.148 | 0.017 | 0.612 |  | 0.068 | -0.152 | 0.289 | **0.112** | 0.018 | 0.543 |
|  | Recruitment Centre (Mannheim) | **-0.370** | **-0.645** | **-0.096** | **0.140** | **-0.078** | **0.008** |  | **-0.325** | **-0.562** | **-0.089** | **0.116** | **-0.080** | **0.007** |
|  | Recruitment Centre (Nottingham) | **0.423** | **0.146** | **0.701** | **0.142** | **0.112** | **0.003** |  | **0.366** | **0.133** | **0.598** | **0.118** | **0.104** | **0.002** |
|  | Recruitment Centre (Paris) | -0.042 | -0.369 | 0.284 | 0.167 | -0.010 | 0.799 |  | -0.016 | -0.267 | 0.235 | 0.128 | -0.004 | 0.899 |
|  | Mean PDS score | -0.093 | -0.208 | 0.022 | 0.059 | -0.034 | 0.111 |  | -0.060 | -0.191 | 0.072 | 0.067 | -0.020 | 0.375 |
|  | Whole Brain Volume | **9.140** | **8.556** | **9.725** | **0.298** | **0.676** | **<0.001** |  | **8.783** | **8.172** | **9.395** | **0.307** | **0.633** | **<0.001** |
|  | Family Support | 0.045 | -0.029 | 0.118 | 0.038 | 0.035 | 0.238 |  | -0.012 | -0.090 | 0.067 | 0.040 | -0.010 | 0.774 |
|  | Peer Problems | 0.010 | -0.074 | 0.093 | 0.043 | 0.008 | 0.817 |  | -0.025 | -0.102 | 0.053 | 0.039 | -0.021 | 0.530 |
|  | Socioeconomic Stress | -0.018 | -0.121 | 0.085 | 0.053 | -0.012 | 0.732 |  | -0.057 | -0.145 | 0.030 | 0.044 | -0.044 | 0.196 |
| Covariance | Amygdala and vmPFC | 0.003 | -0.021 | 0.027 | 0.012 | 0.008 | 0.807 |  | -0.008 | -0.025 | 0.009 | 0.009 | -0.026 | 0.372 |

Note: Statistically significant values (p < .05) are in bold for ease of reading. For recruitment centre, the reference category is Berlin. WBV values were divided by 1,000,000. Amygdala and vmPFC GMV values were divided by 1,000.

**S5 Table. Regression statistics for sensitivity analysis with the inclusion of parental education.**

|  |  |  |  | **Males (n = 948)** | | | |  | **Females (n = 990)** | | | | | | |
| --- | --- | --- | --- | --- | --- | --- | --- | --- | --- | --- | --- | --- | --- | --- | --- |
| **Outcome** | **Predictor** | **Estimate** | **CI lower** | **CI upper** | **SE** | **β** | **p-value** |  | **Estimate** | **CI lower** | **CI upper** | **SE** | **β** | **p-value** |  |
| Socioeconomic Stress | Parental Education | **-0.070** | **-0.097** | **-0.043** | **0.014** | **-0.250** | **<0.001** |  | **-0.074** | **-0.103** | **-0.044** | **0.015** | **-0.241** | **<0.001** |  |
| Emotional Symptoms | Psychiatric Diagnosis (Yes) | 0.238 | -0.097 | 0.572 | 0.171 | 0.058 | 0.163 |  | **0.755** | **0.470** | **1.040** | **0.145** | **0.214** | **<0.001** |  |
|  | Parental Education | 0.020 | -0.013 | 0.053 | 0.017 | 0.052 | 0.234 |  | 0.016 | -0.015 | 0.047 | 0.016 | 0.043 | 0.312 |  |
|  | Recruitment Centre (Dresden) | -0.347 | -0.775 | 0.081 | 0.218 | -0.090 | 0.112 |  | 0.273 | -0.117 | 0.664 | 0.199 | 0.071 | 0.170 |  |
|  | Recruitment Centre (Dublin) | 0.281 | -0.217 | 0.778 | 0.254 | 0.065 | 0.269 |  | **0.471** | **0.060** | **0.881** | **0.209** | **0.105** | **0.025** |  |
|  | Recruitment Centre (Hamburg) | -0.275 | -0.685 | 0.134 | 0.209 | -0.068 | 0.188 |  | 0.209 | -0.158 | 0.576 | 0.187 | 0.056 | 0.264 |  |
|  | Recruitment Centre (London) | **0.554** | **0.125** | **0.982** | **0.218** | **0.136** | **0.011** |  | 0.229 | -0.145 | 0.602 | 0.190 | 0.061 | 0.230 |  |
|  | Recruitment Centre (Mannheim) | -0.450 | -0.917 | 0.017 | 0.238 | -0.107 | 0.059 |  | -0.143 | -0.559 | 0.274 | 0.212 | -0.036 | 0.502 |  |
|  | Recruitment Centre (Nottingham) | 0.286 | -0.107 | 0.679 | 0.201 | 0.085 | 0.154 |  | -0.030 | -0.387 | 0.327 | 0.182 | -0.009 | 0.869 |  |
|  | Recruitment Centre (Paris) | 0.204 | -0.251 | 0.658 | 0.232 | 0.053 | 0.380 |  | 0.216 | -0.207 | 0.640 | 0.216 | 0.057 | 0.316 |  |
|  | Mean PDS score | -0.111 | -0.298 | 0.077 | 0.096 | -0.045 | 0.247 |  | -0.071 | -0.300 | 0.157 | 0.117 | -0.024 | 0.541 |  |
|  | Family Support | 0.005 | -0.117 | 0.127 | 0.062 | 0.005 | 0.932 |  | -0.120 | -0.246 | 0.006 | 0.064 | -0.105 | 0.062 |  |
|  | Peer Problems | **0.722** | **0.574** | **0.869** | **0.075** | **0.623** | **<0.001** |  | **0.566** | **0.404** | **0.729** | **0.083** | **0.494** | **<0.001** |  |
|  | Socioeconomic Stress | -0.144 | -0.301 | 0.013 | 0.080 | -0.105 | 0.071 |  | 0.001 | -0.139 | 0.141 | 0.072 | 0.001 | 0.989 |  |
|  | Whole Brain Volume | 1.123 | -0.406 | 2.652 | 0.780 | 0.093 | 0.150 |  | -0.253 | -1.769 | 1.263 | 0.774 | -0.019 | 0.744 |  |
|  | Amygdala GMV | 0.066 | -0.203 | 0.334 | 0.137 | 0.022 | 0.631 |  | -0.013 | -0.284 | 0.258 | 0.138 | -0.004 | 0.925 |  |
|  | vmPFC GMV | **-0.124** | **-0.227** | **-0.021** | **0.053** | **-0.139** | **0.019** |  | 0.016 | -0.083 | 0.115 | 0.05 | 0.017 | 0.745 |  |
| Family Support | Psychiatric Diagnosis (Yes) | **-0.354** | **-0.651** | **-0.057** | **0.152** | **-0.097** | **0.020** |  | **-0.428** | **-0.698** | **-0.158** | **0.138** | **-0.139** | **0.002** |  |
|  | Parental Education | 0.015 | -0.015 | 0.045 | 0.015 | 0.044 | 0.328 |  | **0.039** | **0.009** | **0.068** | **0.015** | **0.118** | **0.010** |  |
|  | Recruitment Centre (Dresden) | 0.055 | -0.322 | 0.432 | 0.192 | 0.016 | 0.773 |  | -0.085 | -0.472 | 0.302 | 0.197 | -0.025 | 0.665 |  |
|  | Recruitment Centre (Dublin) | -0.112 | -0.497 | 0.274 | 0.197 | -0.029 | 0.570 |  | -0.199 | -0.600 | 0.201 | 0.204 | -0.051 | 0.329 |  |
|  | Recruitment Centre (Hamburg) | **0.397** | **0.011** | **0.783** | **0.197** | **0.112** | **0.044** |  | 0.048 | -0.297 | 0.393 | 0.176 | 0.015 | 0.786 |  |
|  | Recruitment Centre (London) | -0.065 | -0.449 | 0.318 | 0.196 | -0.018 | 0.738 |  | -0.295 | -0.653 | 0.064 | 0.183 | -0.090 | 0.107 |  |
|  | Recruitment Centre (Mannheim) | 0.288 | -0.105 | 0.681 | 0.201 | 0.077 | 0.151 |  | -0.116 | -0.495 | 0.263 | 0.193 | -0.033 | 0.550 |  |
|  | Recruitment Centre (Nottingham) | -0.285 | -0.623 | 0.053 | 0.172 | -0.096 | 0.098 |  | **-0.469** | **-0.805** | **-0.133** | **0.172** | **-0.157** | **0.006** |  |
|  | Recruitment Centre (Paris) | **-0.506** | **-0.858** | **-0.154** | **0.180** | **-0.150** | **0.005** |  | **-0.558** | **-0.943** | **-0.174** | **0.196** | **-0.167** | **0.004** |  |
|  | Mean PDS score | 0.101 | -0.075 | 0.277 | 0.090 | 0.047 | 0.261 |  | 0.070 | -0.136 | 0.276 | 0.105 | 0.027 | 0.506 |  |
|  | Peer Problems | -0.076 | -0.191 | 0.038 | 0.058 | -0.075 | 0.190 |  | 0.010 | -0.108 | 0.129 | 0.060 | 0.01 | 0.866 |  |
|  | Socioeconomic Stress | **-0.213** | **-0.341** | **-0.085** | **0.065** | **-0.177** | **0.001** |  | **-0.336** | **-0.461** | **-0.211** | **0.064** | **-0.314** | **<0.001** |  |
| Peer Problems | Psychiatric Diagnosis (Yes) | **0.786** | **0.490** | **1.082** | **0.151** | **0.221** | **<0.001** |  | **0.902** | **0.636** | **1.168** | **0.136** | **0.293** | **<0.001** |  |
|  | Parental Education | -0.025 | -0.055 | 0.004 | 0.015 | -0.076 | 0.096 |  | -0.014 | -0.045 | 0.016 | 0.015 | -0.043 | 0.357 |  |
|  | Recruitment Centre (Dresden) | **0.487** | **0.109** | **0.866** | **0.193** | **0.147** | **0.012** |  | **0.580** | **0.216** | **0.944** | **0.186** | **0.172** | **0.002** |  |
|  | Recruitment Centre (Dublin) | **-0.457** | **-0.846** | **-0.068** | **0.198** | **-0.122** | **0.021** |  | -0.344 | -0.752 | 0.064 | 0.208 | -0.087 | 0.099 |  |
|  | Recruitment Centre (Hamburg) | 0.046 | -0.330 | 0.422 | 0.192 | 0.013 | 0.811 |  | 0.101 | -0.267 | 0.469 | 0.188 | 0.031 | 0.591 |  |
|  | Recruitment Centre (London) | -0.169 | -0.538 | 0.201 | 0.188 | -0.048 | 0.371 |  | 0.295 | -0.063 | 0.654 | 0.183 | 0.090 | 0.107 |  |
|  | Recruitment Centre (Mannheim) | 0.163 | -0.246 | 0.571 | 0.208 | 0.045 | 0.435 |  | 0.231 | -0.134 | 0.597 | 0.186 | 0.066 | 0.215 |  |
|  | Recruitment Centre (Nottingham) | 0.059 | -0.288 | 0.405 | 0.177 | 0.020 | 0.740 |  | -0.013 | -0.356 | 0.330 | 0.175 | -0.004 | 0.941 |  |
|  | Recruitment Centre (Paris) | **-0.524** | **-0.926** | **-0.123** | **0.205** | **-0.159** | **0.010** |  | **-0.510** | **-0.904** | **-0.116** | **0.201** | **-0.153** | **0.011** |  |
|  | Mean PDS score | -0.066 | -0.243 | 0.111 | 0.090 | -0.031 | 0.465 |  | 0.091 | -0.129 | 0.312 | 0.112 | 0.035 | 0.416 |  |
|  | Socioeconomic Stress | 0.047 | -0.099 | 0.192 | 0.074 | 0.039 | 0.530 |  | 0.091 | -0.026 | 0.208 | 0.060 | 0.085 | 0.129 |  |
| Whole Brain Volume | Psychiatric Diagnosis (Yes) | **-0.026** | **-0.046** | **-0.005** | **0.010** | **-0.075** | **0.015** |  | -0.003 | -0.021 | 0.015 | 0.009 | -0.013 | 0.711 |  |
|  | Parental Education | **0.005** | **0.002** | **0.007** | **0.001** | **0.148** | **<0.001** |  | **0.003** | **0.001** | **0.005** | **0.001** | **0.109** | **0.002** |  |
|  | Recruitment Centre (Dresden) | **-0.051** | **-0.078** | **-0.023** | **0.014** | **-0.160** | **<0.001** |  | -0.005 | -0.028 | 0.019 | 0.012 | -0.017 | 0.696 |  |
|  | Recruitment Centre (Dublin) | **0.049** | **0.017** | **0.081** | **0.016** | **0.136** | **0.003** |  | **0.062** | **0.038** | **0.087** | **0.013** | **0.187** | **<0.001** |  |
|  | Recruitment Centre (Hamburg) | **-0.034** | **-0.063** | **-0.006** | **0.015** | **-0.103** | **0.019** |  | -0.014 | -0.036 | 0.009 | 0.012 | -0.049 | 0.246 |  |
|  | Recruitment Centre (London) | 0.021 | -0.008 | 0.049 | 0.015 | 0.062 | 0.154 |  | 0.008 | -0.014 | 0.031 | 0.012 | 0.030 | 0.478 |  |
|  | Recruitment Centre (Mannheim) | **-0.034** | **-0.061** | **-0.007** | **0.014** | **-0.097** | **0.015** |  | -0.023 | -0.046 | 0.000 | 0.012 | -0.076 | 0.054 |  |
|  | Recruitment Centre (Nottingham) | 0.005 | -0.022 | 0.032 | 0.014 | 0.017 | 0.734 |  | 0.015 | -0.007 | 0.038 | 0.011 | 0.061 | 0.177 |  |
|  | Recruitment Centre (Paris) | 0.010 | -0.021 | 0.041 | 0.016 | 0.031 | 0.540 |  | 0.003 | -0.022 | 0.028 | 0.013 | 0.011 | 0.801 |  |
|  | Mean PDS score | **0.016** | **0.004** | **0.029** | **0.006** | **0.081** | **0.008** |  | 0.009 | -0.006 | 0.023 | 0.007 | 0.040 | 0.231 |  |
|  | Family Support | 0.005 | -0.002 | 0.012 | 0.004 | 0.054 | 0.170 |  | 0.002 | -0.005 | 0.010 | 0.004 | 0.029 | 0.521 |  |
|  | Peer Problems | 0.003 | -0.006 | 0.011 | 0.004 | 0.027 | 0.547 |  | -0.002 | -0.010 | 0.005 | 0.004 | -0.029 | 0.523 |  |
|  | Socioeconomic Stress | -0.003 | -0.012 | 0.007 | 0.005 | -0.023 | 0.607 |  | **-0.010** | **-0.018** | **-0.001** | **0.004** | **-0.105** | **0.027** |  |
| Amygdala GMV | Psychiatric Diagnosis (Yes) | 0.026 | -0.055 | 0.108 | 0.042 | 0.019 | 0.526 |  | **-0.073** | **-0.141** | **-0.004** | **0.035** | **-0.063** | **0.038** |  |
|  | Parental Education | -0.003 | -0.012 | 0.005 | 0.004 | -0.025 | 0.464 |  | 0.005 | -0.002 | 0.012 | 0.004 | 0.040 | 0.162 |  |
|  | Recruitment Centre (Dresden) | 0.059 | -0.045 | 0.163 | 0.053 | 0.045 | 0.270 |  | 0.029 | -0.063 | 0.120 | 0.047 | 0.023 | 0.535 |  |
|  | Recruitment Centre (Dublin) | -0.033 | -0.150 | 0.084 | 0.060 | -0.023 | 0.581 |  | **-0.098** | **-0.190** | **-0.006** | **0.047** | **-0.066** | **0.037** |  |
|  | Recruitment Centre (Hamburg) | **0.117** | **0.006** | **0.228** | **0.057** | **0.086** | **0.039** |  | 0.004 | -0.084 | 0.092 | 0.045 | 0.003 | 0.926 |  |
|  | Recruitment Centre (London) | **-0.133** | **-0.241** | **-0.025** | **0.055** | **-0.097** | **0.016** |  | **-0.297** | **-0.386** | **-0.207** | **0.046** | **-0.241** | **<0.001** |  |
|  | Recruitment Centre (Mannheim) | 0.052 | -0.042 | 0.146 | 0.048 | 0.037 | 0.276 |  | -0.060 | -0.147 | 0.026 | 0.044 | -0.046 | 0.172 |  |
|  | Recruitment Centre (Nottingham) | **0.210** | **0.120** | **0.300** | **0.046** | **0.185** | **<0.001** |  | **0.177** | **0.097** | **0.257** | **0.041** | **0.158** | **<0.001** |  |
|  | Recruitment Centre (Paris) | 0.048 | -0.058 | 0.154 | 0.054 | 0.037 | 0.374 |  | -0.005 | -0.094 | 0.085 | 0.046 | -0.004 | 0.918 |  |
|  | Mean PDS score | 0.034 | -0.016 | 0.083 | 0.025 | 0.041 | 0.183 |  | 0.037 | -0.018 | 0.092 | 0.028 | 0.038 | 0.189 |  |
|  | Whole Brain Volume | **1.820** | **1.593** | **2.047** | **0.116** | **0.446** | **<0.001** |  | **2.313** | **2.098** | **2.529** | **0.110** | **0.522** | **<0.001** |  |
|  | Family Support | -0.007 | -0.035 | 0.020 | 0.014 | -0.019 | 0.596 |  | -0.001 | -0.028 | 0.025 | 0.013 | -0.004 | 0.918 |  |
|  | Peer Problems | -0.010 | -0.044 | 0.023 | 0.017 | -0.027 | 0.540 |  | 0.025 | -0.004 | 0.054 | 0.015 | 0.066 | 0.093 |  |
|  | Socioeconomic Stress | -0.034 | -0.074 | 0.005 | 0.02 | -0.075 | 0.086 |  | 0.001 | -0.031 | 0.032 | 0.016 | 0.001 | 0.982 |  |
| vmPFC GMV | Psychiatric Diagnosis (Yes) | 0.134 | -0.085 | 0.352 | 0.112 | 0.029 | 0.231 |  | -0.049 | -0.233 | 0.135 | 0.094 | -0.014 | 0.602 |  |
|  | Parental Education | 0.021 | -0.002 | 0.043 | 0.011 | 0.048 | 0.071 |  | -0.012 | -0.031 | 0.007 | 0.010 | -0.031 | 0.207 |  |
|  | Recruitment Centre (Dresden) | -0.261 | -0.557 | 0.034 | 0.151 | -0.061 | 0.083 |  | -0.116 | -0.372 | 0.139 | 0.130 | -0.029 | 0.371 |  |
|  | Recruitment Centre (Dublin) | **0.337** | **0.030** | **0.645** | **0.157** | **0.070** | **0.031** |  | **0.669** | **0.430** | **0.908** | **0.122** | **0.145** | **<0.001** |  |
|  | Recruitment Centre (Hamburg) | 0.173 | -0.131 | 0.476 | 0.155 | 0.038 | 0.265 |  | -0.019 | -0.252 | 0.214 | 0.119 | -0.005 | 0.871 |  |
|  | Recruitment Centre (London) | 0.050 | -0.241 | 0.341 | 0.148 | 0.011 | 0.735 |  | 0.072 | -0.152 | 0.296 | 0.114 | 0.019 | 0.527 |  |
|  | Recruitment Centre (Mannheim) | **-0.377** | **-0.654** | **-0.099** | **0.142** | **-0.080** | **0.008** |  | **-0.316** | **-0.556** | **-0.076** | **0.122** | **-0.077** | **0.010** |  |
|  | Recruitment Centre (Nottingham) | **0.416** | **0.139** | **0.693** | **0.141** | **0.110** | **0.003** |  | **0.380** | **0.147** | **0.612** | **0.119** | **0.108** | **0.001** |  |
|  | Recruitment Centre (Paris) | -0.085 | -0.423 | 0.254 | 0.173 | -0.020 | 0.624 |  | 0.019 | -0.243 | 0.282 | 0.134 | 0.005 | 0.884 |  |
|  | Mean PDS score | -0.089 | -0.206 | 0.028 | 0.060 | -0.033 | 0.134 |  | -0.055 | -0.184 | 0.075 | 0.066 | -0.018 | 0.408 |  |
|  | Whole Brain Volume | **9.050** | **8.470** | **9.630** | **0.296** | **0.669** | **<0.001** |  | **8.802** | **8.185** | **9.418** | **0.315** | **0.636** | **<0.001** |  |
|  | Family Support | 0.042 | -0.031 | 0.116 | 0.037 | 0.033 | 0.257 |  | -0.002 | -0.081 | 0.076 | 0.040 | -0.002 | 0.954 |  |
|  | Peer Problems | 0.014 | -0.069 | 0.096 | 0.042 | 0.010 | 0.747 |  | -0.021 | -0.099 | 0.057 | 0.040 | -0.018 | 0.593 |  |
|  | Socioeconomic Stress | -0.004 | -0.109 | 0.101 | 0.054 | -0.002 | 0.944 |  | -0.053 | -0.136 | 0.029 | 0.042 | -0.042 | 0.207 |  |
| Covariance | Amygdala and vmPFC | 0.003 | -0.020 | 0.027 | 0.012 | 0.008 | 0.793 |  | -0.010 | -0.027 | 0.008 | 0.009 | -0.032 | 0.282 |  |

Note: Statistically significant values (p < .05) are in bold for ease of reading. For recruitment centre, the reference category is Berlin. WBV values were divided by 1,000,000. Amygdala and vmPFC GMV values were divided by 1,000.

**S6 Table. Distribution of psychiatric diagnoses by sex, separately for DSM-IV and ICD-10.**

|  | **DSM-IV** |  |  | **ICD-10** |  |  |
| --- | --- | --- | --- | --- | --- | --- |
| **Diagnosis** | **Male n** | **Female n** | **Total n** | **Male n** | **Female n** | **Total n** |
| ADHD/Autism | 44 | 12 | 56 | 35 | 11 | 46 |
| Mood Disorder | 32 | 96 | 128 | 33 | 95 | 128 |
| Anxiety Disorder | 17 | 62 | 79 | 18 | 62 | 80 |
| Conduct/Oppositional Disorder | 29 | 33 | 62 | 31 | 30 | 61 |
| Other Disorder | 11 | 23 | 34 | 12 | 27 | 39 |

Note: ADHD/Autism: ADHD Combined, ADHD Hyperactive, ADHD Impulsive, ADHD Other, ADHD Any, PDD/Autism; Mood Disorder: Emotional disorder, Major depression, Mania/Bipolar, Other depression; Anxiety Disorder: Agoraphobia, Generalised anxiety disorder, OCD, Other anxiety disorder, Panic disorder, PTSD, Separation anxiety, Social phobia, Specific phobia; Conduct/Oppositional Disorder: Any Conduct/Oppositional Disorder, Conduct disorder, Oppositional defiant disorder, Other disruptive disorder; Other Disorder: Other disorder, Eating disorder, Tic disorder. Some participants had more than one diagnosis, which is why the total does not equal the total of the psychiatric diagnosis variable.

**S7 Table. Regression statistics for the sensitivity analysis of the exclusion of participants with a psychiatric diagnosis.**

|  |  | **Males (n = 843)** | | | | |  | | **Females (n = 841)** | | | | | |  |
| --- | --- | --- | --- | --- | --- | --- | --- | --- | --- | --- | --- | --- | --- | --- | --- |
| **Outcome** | **Predictor** | **Estimate** | **CI lower** | **CI upper** | **SE** | **β** | **p-value** |  | **Estimate** | **CI lower** | **CI upper** | **SE** | **β** | **p-value** | |
| Socioeconomic Stress | Parental Education | **-0.069** | **-0.099** | **-0.039** | **0.015** | **-0.234** | **<0.001** |  | **-0.075** | **-0.108** | **-0.042** | **0.017** | **-0.243** | **<0.001** | |
| Emotional Symptoms | Parental Education | 0.028 | -0.007 | 0.064 | 0.018 | 0.077 | 0.117 |  | 0.017 | -0.017 | 0.051 | 0.017 | 0.048 | 0.326 | |
|  | Recruitment Centre (Dresden) | -0.429 | -0.881 | 0.022 | 0.230 | -0.122 | 0.062 |  | 0.219 | -0.208 | 0.647 | 0.218 | 0.064 | 0.314 | |
|  | Recruitment Centre (Dublin) | 0.342 | -0.222 | 0.906 | 0.288 | 0.082 | 0.235 |  | 0.367 | -0.111 | 0.846 | 0.244 | 0.088 | 0.132 | |
|  | Recruitment Centre (Hamburg) | -0.302 | -0.757 | 0.153 | 0.232 | -0.077 | 0.193 |  | 0.224 | -0.191 | 0.640 | 0.212 | 0.063 | 0.290 | |
|  | Recruitment Centre (London) | **0.512** | **0.057** | **0.968** | **0.232** | **0.134** | **0.027** |  | 0.030 | -0.399 | 0.458 | 0.219 | 0.009 | 0.892 | |
|  | Recruitment Centre (Mannheim) | **-0.495** | **-0.984** | **-0.006** | **0.249** | **-0.125** | **0.047** |  | -0.219 | -0.697 | 0.258 | 0.244 | -0.059 | 0.368 | |
|  | Recruitment Centre (Nottingham) | 0.200 | -0.229 | 0.628 | 0.219 | 0.063 | 0.361 |  | -0.167 | -0.576 | 0.243 | 0.209 | -0.053 | 0.424 | |
|  | Recruitment Centre (Paris) | 0.182 | -0.341 | 0.705 | 0.267 | 0.048 | 0.495 |  | 0.074 | -0.418 | 0.565 | 0.251 | 0.021 | 0.769 | |
|  | Mean PDS score | -0.126 | -0.329 | 0.076 | 0.104 | -0.055 | 0.222 |  | -0.122 | -0.371 | 0.126 | 0.127 | -0.046 | 0.335 | |
|  | Family Support | -0.012 | -0.144 | 0.121 | 0.068 | -0.010 | 0.864 |  | **-0.213** | **-0.354** | **-0.071** | **0.072** | **-0.196** | **0.003** | |
|  | Peer Problems | **0.666** | **0.501** | **0.831** | **0.084** | **0.618** | **<0.001** |  | **0.537** | **0.357** | **0.717** | **0.092** | **0.496** | **<0.001** | |
|  | Socioeconomic Stress | **-0.187** | **-0.345** | **-0.029** | **0.081** | **-0.150** | **0.020** |  | -0.061 | -0.225 | 0.103 | 0.084 | -0.054 | 0.465 | |
|  | Whole Brain Volume | 0.860 | -0.785 | 2.504 | 0.839 | 0.074 | 0.305 |  | -0.232 | -1.907 | 1.443 | 0.855 | -0.018 | 0.786 | |
|  | Amygdala GMV | -0.003 | -0.286 | 0.280 | 0.145 | -0.001 | 0.984 |  | -0.031 | -0.341 | 0.278 | 0.158 | -0.011 | 0.844 | |
|  | vmPFC GMV | **-0.116** | **-0.226** | **-0.005** | **0.056** | **-0.136** | **0.040** |  | -0.011 | -0.119 | 0.097 | 0.055 | -0.012 | 0.842 | |
| Family Support | Parental Education | 0.017 | -0.014 | 0.049 | 0.016 | 0.052 | 0.276 |  | **0.032** | **0.001** | **0.064** | **0.016** | **0.100** | **0.045** | |
|  | Recruitment Centre (Dresden) | -0.041 | -0.428 | 0.347 | 0.198 | -0.013 | 0.837 |  | -0.089 | -0.511 | 0.334 | 0.215 | -0.028 | 0.681 | |
|  | Recruitment Centre (Dublin) | -0.204 | -0.614 | 0.206 | 0.209 | -0.054 | 0.330 |  | -0.319 | -0.774 | 0.136 | 0.232 | -0.082 | 0.170 | |
|  | Recruitment Centre (Hamburg) | 0.268 | -0.150 | 0.686 | 0.213 | 0.075 | 0.209 |  | 0.085 | -0.325 | 0.494 | 0.209 | 0.026 | 0.685 | |
|  | Recruitment Centre (London) | -0.211 | -0.611 | 0.188 | 0.204 | -0.061 | 0.300 |  | -0.379 | -0.795 | 0.038 | 0.213 | -0.118 | 0.075 | |
|  | Recruitment Centre (Mannheim) | 0.172 | -0.240 | 0.584 | 0.210 | 0.048 | 0.414 |  | -0.062 | -0.493 | 0.369 | 0.220 | -0.018 | 0.778 | |
|  | Recruitment Centre (Nottingham) | -0.336 | -0.691 | 0.020 | 0.181 | -0.116 | 0.064 |  | **-0.471** | **-0.857** | **-0.085** | **0.197** | **-0.161** | **0.017** | |
|  | Recruitment Centre (Paris) | **-0.799** | **-1.179** | **-0.419** | **0.194** | **-0.232** | **<0.001** |  | **-0.563** | **-0.998** | **-0.127** | **0.222** | **-0.170** | **0.011** | |
|  | Mean PDS score | 0.090 | -0.096 | 0.275 | 0.095 | 0.043 | 0.344 |  | 0.060 | -0.160 | 0.280 | 0.112 | 0.024 | 0.594 | |
|  | Peer Problems | -0.077 | -0.196 | 0.042 | 0.061 | -0.079 | 0.202 |  | 0.049 | -0.088 | 0.187 | 0.070 | 0.049 | 0.483 | |
|  | Socioeconomic Stress | **-0.190** | **-0.316** | **-0.063** | **0.065** | **-0.167** | **0.003** |  | **-0.317** | **-0.462** | **-0.173** | **0.074** | **-0.302** | **<0.001** | |
| Peer Problems | Parental Education | -0.026 | -0.059 | 0.007 | 0.017 | -0.076 | 0.122 |  | -0.002 | -0.036 | 0.032 | 0.017 | -0.006 | 0.915 | |
|  | Recruitment Centre (Dresden) | **0.458** | **0.037** | **0.880** | **0.215** | **0.140** | **0.033** |  | **0.598** | **0.184** | **1.011** | **0.211** | **0.190** | **0.005** | |
|  | Recruitment Centre (Dublin) | **-0.669** | **-1.113** | **-0.225** | **0.227** | **-0.174** | **0.003** |  | **-0.515** | **-0.976** | **-0.053** | **0.235** | **-0.133** | **0.029** | |
|  | Recruitment Centre (Hamburg) | 0.043 | -0.397 | 0.482 | 0.224 | 0.012 | 0.849 |  | 0.098 | -0.342 | 0.539 | 0.225 | 0.030 | 0.662 | |
|  | Recruitment Centre (London) | -0.307 | -0.717 | 0.103 | 0.209 | -0.087 | 0.142 |  | **0.445** | **0.025** | **0.864** | **0.214** | **0.139** | **0.038** | |
|  | Recruitment Centre (Mannheim) | 0.147 | -0.303 | 0.597 | 0.229 | 0.040 | 0.522 |  | 0.335 | -0.088 | 0.759 | 0.216 | 0.098 | 0.121 | |
|  | Recruitment Centre (Nottingham) | -0.007 | -0.398 | 0.385 | 0.200 | -0.002 | 0.974 |  | 0.033 | -0.371 | 0.436 | 0.206 | 0.011 | 0.873 | |
|  | Recruitment Centre (Paris) | **-0.696** | **-1.163** | **-0.230** | **0.238** | **-0.199** | **0.003** |  | **-0.491** | **-0.970** | **-0.012** | **0.244** | **-0.148** | **0.045** | |
|  | Mean PDS score | 0.052 | -0.142 | 0.247 | 0.099 | 0.024 | 0.599 |  | 0.217 | -0.028 | 0.463 | 0.125 | 0.087 | 0.083 | |
|  | Socioeconomic Stress | 0.148 | -0.007 | 0.303 | 0.079 | 0.128 | 0.061 |  | **0.159** | **0.019** | **0.299** | **0.072** | **0.151** | **0.026** | |
| Whole Brain Volume | Parental Education | **0.005** | **0.002** | **0.007** | **0.001** | **0.149** | **<0.001** |  | **0.002** | **0.000** | **0.004** | **0.001** | **0.077** | **0.039** | |
|  | Recruitment Centre (Dresden) | **-0.054** | **-0.082** | **-0.026** | **0.014** | **-0.178** | **<0.001** |  | 0.002 | -0.024 | 0.027 | 0.013 | 0.006 | 0.901 | |
|  | Recruitment Centre (Dublin) | **0.047** | **0.013** | **0.080** | **0.017** | **0.131** | **0.006** |  | **0.070** | **0.040** | **0.100** | **0.015** | **0.210** | **<0.001** | |
|  | Recruitment Centre (Hamburg) | **-0.031** | **-0.062** | **-0.001** | **0.015** | **-0.094** | **0.041** |  | -0.006 | -0.031 | 0.019 | 0.013 | -0.022 | 0.638 | |
|  | Recruitment Centre (London) | 0.012 | -0.018 | 0.042 | 0.015 | 0.038 | 0.420 |  | 0.008 | -0.017 | 0.033 | 0.013 | 0.028 | 0.549 | |
|  | Recruitment Centre (Mannheim) | **-0.040** | **-0.068** | **-0.012** | **0.014** | **-0.118** | **0.005** |  | -0.020 | -0.045 | 0.005 | 0.013 | -0.070 | 0.109 | |
|  | Recruitment Centre (Nottingham) | 0.004 | -0.025 | 0.032 | 0.015 | 0.014 | 0.796 |  | 0.020 | -0.005 | 0.044 | 0.012 | 0.078 | 0.115 | |
|  | Recruitment Centre (Paris) | 0.010 | -0.024 | 0.044 | 0.017 | 0.031 | 0.560 |  | 0.017 | -0.012 | 0.046 | 0.015 | 0.060 | 0.256 | |
|  | Mean PDS score | **0.019** | **0.007** | **0.032** | **0.006** | **0.098** | **0.002** |  | 0.013 | -0.003 | 0.028 | 0.008 | 0.060 | 0.104 | |
|  | Family Support | 0.004 | -0.004 | 0.011 | 0.004 | 0.040 | 0.340 |  | 0.001 | -0.008 | 0.008 | 0.004 | 0.003 | 0.943 | |
|  | Peer Problems | 0.005 | -0.004 | 0.014 | 0.005 | 0.051 | 0.315 |  | 0.001 | -0.009 | 0.008 | 0.004 | -0.002 | 0.964 | |
|  | Socioeconomic Stress | -0.006 | -0.016 | 0.004 | 0.005 | -0.054 | 0.248 |  | **-0.015** | **-0.024** | **-0.005** | **0.005** | **-0.164** | **0.002** | |
| Amygdala GMV | Parental Education | -0.001 | -0.010 | 0.008 | 0.005 | -0.006 | 0.860 |  | 0.006 | -0.002 | 0.013 | 0.004 | 0.046 | 0.129 | |
|  | Recruitment Centre (Dresden) | 0.072 | -0.036 | 0.181 | 0.055 | 0.058 | 0.191 |  | 0.039 | -0.063 | 0.140 | 0.052 | 0.032 | 0.457 | |
|  | Recruitment Centre (Dublin) | 0.003 | -0.124 | 0.130 | 0.065 | 0.002 | 0.960 |  | -0.091 | -0.193 | 0.010 | 0.052 | -0.062 | 0.079 | |
|  | Recruitment Centre (Hamburg) | **0.124** | **0.006** | **0.242** | **0.060** | **0.089** | **0.039** |  | 0.012 | -0.089 | 0.112 | 0.051 | 0.009 | 0.818 | |
|  | Recruitment Centre (London) | -0.109 | -0.226 | 0.007 | 0.059 | -0.080 | 0.066 |  | **-0.283** | **-0.383** | **-0.183** | **0.051** | **-0.231** | **<0.001** | |
|  | Recruitment Centre (Mannheim) | 0.066 | -0.036 | 0.167 | 0.052 | 0.047 | 0.204 |  | -0.055 | -0.152 | 0.043 | 0.050 | -0.042 | 0.271 | |
|  | Recruitment Centre (Nottingham) | **0.230** | **0.134** | **0.327** | **0.049** | **0.203** | **<0.001** |  | **0.179** | **0.088** | **0.269** | **0.046** | **0.160** | **<0.001** | |
|  | Recruitment Centre (Paris) | 0.100 | -0.018 | 0.219 | 0.060 | 0.074 | 0.097 |  | -0.010 | -0.121 | 0.102 | 0.057 | -0.008 | 0.863 | |
|  | Mean PDS score | 0.046 | -0.008 | 0.099 | 0.027 | 0.056 | 0.093 |  | 0.047 | -0.013 | 0.107 | 0.030 | 0.050 | 0.121 | |
|  | Whole Brain Volume | **1.787** | **1.543** | **2.032** | **0.125** | **0.430** | **<0.001** |  | **2.289** | **2.041** | **2.536** | **0.126** | **0.515** | **<0.001** | |
|  | Family Support | -0.007 | -0.036 | 0.022 | 0.015 | -0.018 | 0.641 |  | -0.008 | -0.037 | 0.020 | 0.015 | -0.022 | 0.562 | |
|  | Peer Problems | -0.006 | -0.042 | 0.029 | 0.018 | -0.017 | 0.723 |  | 0.026 | -0.009 | 0.061 | 0.018 | 0.069 | 0.145 | |
|  | Socioeconomic Stress | -0.037 | -0.077 | 0.003 | 0.020 | -0.082 | 0.073 |  | -0.008 | -0.045 | 0.028 | 0.019 | -0.021 | 0.651 | |
| vmPFC GMV | Parental Education | 0.019 | -0.004 | 0.042 | 0.012 | 0.044 | 0.110 |  | -0.008 | -0.028 | 0.012 | 0.010 | -0.020 | 0.450 | |
|  | Recruitment Centre (Dresden) | -0.240 | -0.538 | 0.058 | 0.152 | -0.058 | 0.115 |  | -0.100 | -0.376 | 0.177 | 0.141 | -0.027 | 0.480 | |
|  | Recruitment Centre (Dublin) | **0.417** | **0.099** | **0.735** | **0.162** | **0.086** | **0.010** |  | **0.683** | **0.392** | **0.973** | **0.148** | **0.148** | **<0.001** | |
|  | Recruitment Centre (Hamburg) | 0.139 | -0.174 | 0.451 | 0.160 | 0.030 | 0.385 |  | 0.004 | -0.251 | 0.258 | 0.130 | 0.001 | 0.976 | |
|  | Recruitment Centre (London) | 0.094 | -0.202 | 0.390 | 0.151 | 0.021 | 0.533 |  | 0.053 | -0.192 | 0.298 | 0.125 | 0.014 | 0.670 | |
|  | Recruitment Centre (Mannheim) | **-0.357** | **-0.641** | **-0.072** | **0.145** | **-0.077** | **0.014** |  | **-0.310** | **-0.569** | **-0.051** | **0.132** | **-0.076** | **0.019** | |
|  | Recruitment Centre (Nottingham) | **0.416** | **0.131** | **0.702** | **0.146** | **0.112** | **0.004** |  | **0.398** | **0.143** | **0.652** | **0.130** | **0.114** | **0.002** | |
|  | Recruitment Centre (Paris) | -0.027 | -0.388 | 0.334 | 0.184 | -0.006 | 0.882 |  | 0.031 | -0.279 | 0.340 | 0.158 | 0.008 | 0.846 | |
|  | Mean PDS score | -0.109 | -0.228 | 0.011 | 0.061 | -0.040 | 0.075 |  | -0.045 | -0.187 | 0.098 | 0.073 | -0.015 | 0.539 | |
|  | Whole Brain Volume | **9.097** | **8.439** | **9.755** | **0.336** | **0.665** | **<0.001** |  | **8.746** | **8.071** | **9.422** | **0.344** | **0.631** | **<0.001** | |
|  | Family Support | 0.063 | -0.014 | 0.139 | 0.039 | 0.049 | 0.108 |  | -0.037 | -0.122 | 0.049 | 0.044 | -0.031 | 0.404 | |
|  | Peer Problems | 0.030 | -0.061 | 0.121 | 0.046 | 0.024 | 0.514 |  | -0.023 | -0.113 | 0.067 | 0.046 | -0.019 | 0.616 | |
|  | Socioeconomic Stress | -0.003 | -0.110 | 0.105 | 0.055 | -0.002 | 0.959 |  | -0.036 | -0.126 | 0.053 | 0.046 | -0.029 | 0.427 | |
| Covariance | Amygdala and vmPFC | 0.003 | -0.022 | 0.028 | 0.013 | 0.007 | 0.825 |  | -0.007 | -0.027 | 0.013 | 0.010 | -0.023 | 0.509 | |

Note: Statistically significant values (p < .05) are in bold for ease of reading. For recruitment centre, the reference category is Berlin. WBV values were divided by 1,000,000. Amygdala and vmPFC GMV values were divided by 1,000. Model fit: robust χ^2^ = 955.056, p-value < 0.001, robust CFI = 0.922, robust RMSEA = 0.025 [0.022, 0.028].

**S8 Table. Regression statistics for sensitivity analysis of the inclusion of mood or anxiety disorder instead of any psychiatric disorder.**

|  |  | **Males (n = 948)** | | | | | |  | **Females (n = 990)** | | | | | | |
| --- | --- | --- | --- | --- | --- | --- | --- | --- | --- | --- | --- | --- | --- | --- | --- |
| **Outcome** | **Predictor** | **Estimate** | **CI lower** | **CI upper** | **SE** | **β** | **p-value** |  | **Estimate** | **CI lower** | **CI upper** | **SE** | **β** | **p-value** |  |
| Socioeconomic Stress | Parental Education | **-0.070** | **-0.097** | **-0.043** | **0.014** | **-0.250** | **<0.001** |  | **-0.075** | **-0.104** | **-0.046** | **0.015** | **-0.244** | **<0.001** |  |
| Emotional Symptoms | Mood or Anxiety Disorder (Yes) | **0.822** | **0.306** | **1.338** | **0.263** | **0.114** | **0.002** |  | **1.367** | **1.040** | **1.694** | **0.167** | **0.312** | **<0.001** |  |
|  | Parental Education | 0.017 | -0.017 | 0.051 | 0.018 | 0.043 | 0.334 |  | 0.018 | -0.013 | 0.050 | 0.016 | 0.047 | 0.252 |  |
|  | Recruitment Centre (Dresden) | -0.333 | -0.765 | 0.098 | 0.220 | -0.085 | 0.130 |  | 0.256 | -0.137 | 0.648 | 0.200 | 0.064 | 0.202 |  |
|  | Recruitment Centre (Dublin) | 0.235 | -0.276 | 0.745 | 0.260 | 0.053 | 0.367 |  | 0.396 | -0.025 | 0.816 | 0.215 | 0.085 | 0.065 |  |
|  | Recruitment Centre (Hamburg) | -0.257 | -0.670 | 0.156 | 0.211 | -0.062 | 0.223 |  | 0.184 | -0.187 | 0.556 | 0.189 | 0.048 | 0.331 |  |
|  | Recruitment Centre (London) | **0.525** | **0.084** | **0.966** | **0.225** | **0.126** | **0.020** |  | 0.173 | -0.198 | 0.543 | 0.189 | 0.044 | 0.361 |  |
|  | Recruitment Centre (Mannheim) | -0.421 | -0.895 | 0.052 | 0.242 | -0.098 | 0.081 |  | -0.161 | -0.583 | 0.261 | 0.216 | -0.039 | 0.455 |  |
|  | Recruitment Centre (Nottingham) | 0.254 | -0.146 | 0.654 | 0.204 | 0.074 | 0.214 |  | -0.059 | -0.419 | 0.300 | 0.184 | -0.017 | 0.746 |  |
|  | Recruitment Centre (Paris) | 0.155 | -0.314 | 0.625 | 0.239 | 0.040 | 0.516 |  | 0.131 | -0.298 | 0.561 | 0.219 | 0.033 | 0.549 |  |
|  | Mean PDS score | -0.101 | -0.297 | 0.095 | 0.100 | -0.040 | 0.311 |  | -0.059 | -0.288 | 0.171 | 0.117 | -0.019 | 0.617 |  |
|  | Family Support | 0.003 | -0.122 | 0.129 | 0.064 | 0.003 | 0.960 |  | **-0.154** | **-0.282** | **-0.026** | **0.065** | **-0.129** | **0.018** |  |
|  | Peer Problems | **0.709** | **0.555** | **0.862** | **0.078** | **0.596** | **<0.001** |  | **0.548** | **0.388** | **0.708** | **0.082** | **0.460** | **<0.001** |  |
|  | Socioeconomic Stress | -0.146 | -0.310 | 0.017 | 0.083 | -0.105 | 0.079 |  | -0.008 | -0.150 | 0.134 | 0.072 | -0.006 | 0.914 |  |
|  | Whole Brain Volume | 1.036 | -0.499 | 2.571 | 0.783 | 0.084 | 0.186 |  | -0.196 | -1.737 | 1.345 | 0.786 | -0.014 | 0.803 |  |
|  | Amygdala GMV | 0.085 | -0.185 | 0.355 | 0.138 | 0.028 | 0.537 |  | 0.001 | -0.276 | 0.276 | 0.141 | 0.001 | 0.998 |  |
|  | vmPFC GMV | **-0.123** | **-0.228** | **-0.018** | **0.053** | **-0.135** | **0.021** |  | -0.006 | -0.107 | 0.094 | 0.051 | -0.006 | 0.899 |  |
| Family Support | Mood or Anxiety Disorder (Yes) | -0.084 | -0.635 | 0.468 | 0.281 | -0.014 | 0.766 |  | -0.080 | -0.402 | 0.241 | 0.164 | -0.022 | 0.625 |  |
|  | Parental Education | 0.014 | -0.016 | 0.044 | 0.015 | 0.040 | 0.377 |  | **0.040** | **0.011** | **0.070** | **0.015** | **0.123** | **0.007** |  |
|  | Recruitment Centre (Dresden) | 0.107 | -0.270 | 0.484 | 0.192 | 0.032 | 0.578 |  | 0.008 | -0.372 | 0.387 | 0.194 | 0.002 | 0.969 |  |
|  | Recruitment Centre (Dublin) | -0.111 | -0.492 | 0.270 | 0.194 | -0.029 | 0.567 |  | -0.189 | -0.589 | 0.210 | 0.204 | -0.048 | 0.353 |  |
|  | Recruitment Centre (Hamburg) | **0.397** | **0.009** | **0.784** | **0.198** | **0.112** | **0.045** |  | 0.055 | -0.290 | 0.400 | 0.176 | 0.017 | 0.754 |  |
|  | Recruitment Centre (London) | -0.047 | -0.429 | 0.335 | 0.195 | -0.013 | 0.810 |  | -0.246 | -0.601 | 0.108 | 0.181 | -0.076 | 0.173 |  |
|  | Recruitment Centre (Mannheim) | 0.313 | -0.082 | 0.708 | 0.202 | 0.085 | 0.121 |  | -0.078 | -0.455 | 0.299 | 0.193 | -0.022 | 0.686 |  |
|  | Recruitment Centre (Nottingham) | -0.258 | -0.596 | 0.080 | 0.173 | -0.087 | 0.135 |  | **-0.444** | **-0.776** | **-0.112** | **0.169** | **-0.149** | **0.009** |  |
|  | Recruitment Centre (Paris) | **-0.519** | **-0.870** | **-0.168** | **0.179** | **-0.155** | **0.004** |  | **-0.566** | **-0.952** | **-0.179** | **0.197** | **-0.170** | **0.004** |  |
|  | Mean PDS score | 0.092 | -0.084 | 0.269 | 0.090 | 0.043 | 0.304 |  | 0.046 | -0.159 | 0.251 | 0.105 | 0.018 | 0.658 |  |
|  | Peer Problems | -0.098 | -0.215 | 0.018 | 0.059 | -0.096 | 0.098 |  | -0.034 | -0.153 | 0.086 | 0.061 | -0.034 | 0.580 |  |
|  | Socioeconomic Stress | **-0.226** | **-0.355** | **-0.097** | **0.066** | **-0.189** | **0.001** |  | **-0.344** | **-0.470** | **-0.219** | **0.064** | **-0.323** | **<0.001** |  |
| Peer Problems | Mood or Anxiety Disorder (Yes) | **1.672** | **1.199** | **2.146** | **0.242** | **0.275** | **<0.001** |  | **1.025** | **0.723** | **1.327** | **0.154** | **0.279** | **<0.001** |  |
|  | Parental Education | **-0.030** | **-0.059** | **0.000** | **0.015** | **-0.090** | **0.046** |  | -0.015 | -0.045 | 0.015 | 0.015 | -0.046 | 0.330 |  |
|  | Recruitment Centre (Dresden) | **0.454** | **0.080** | **0.829** | **0.191** | **0.137** | **0.018** |  | **0.486** | **0.127** | **0.844** | **0.183** | **0.144** | **0.008** |  |
|  | Recruitment Centre (Dublin) | **-0.503** | **-0.883** | **-0.123** | **0.194** | **-0.135** | **0.009** |  | **-0.432** | **-0.839** | **-0.026** | **0.208** | **-0.110** | **0.037** |  |
|  | Recruitment Centre (Hamburg) | 0.083 | -0.293 | 0.459 | 0.192 | 0.024 | 0.665 |  | 0.069 | -0.299 | 0.437 | 0.188 | 0.021 | 0.714 |  |
|  | Recruitment Centre (London) | -0.247 | -0.611 | 0.117 | 0.186 | -0.071 | 0.183 |  | 0.219 | -0.137 | 0.575 | 0.182 | 0.067 | 0.229 |  |
|  | Recruitment Centre (Mannheim) | 0.160 | -0.248 | 0.568 | 0.208 | 0.044 | 0.442 |  | 0.189 | -0.171 | 0.549 | 0.184 | 0.054 | 0.303 |  |
|  | Recruitment Centre (Nottingham) | -0.016 | -0.358 | 0.325 | 0.174 | -0.006 | 0.925 |  | -0.046 | -0.387 | 0.296 | 0.174 | -0.015 | 0.793 |  |
|  | Recruitment Centre (Paris) | **-0.559** | **-0.953** | **-0.165** | **0.201** | **-0.170** | **0.005** |  | **-0.561** | **-0.956** | **-0.165** | **0.202** | **-0.168** | **0.005** |  |
|  | Mean PDS score | -0.048 | -0.223 | 0.126 | 0.089 | -0.023 | 0.589 |  | 0.106 | -0.113 | 0.326 | 0.112 | 0.041 | 0.341 |  |
|  | Socioeconomic Stress | 0.057 | -0.089 | 0.203 | 0.075 | 0.048 | 0.446 |  | 0.099 | -0.019 | 0.217 | 0.060 | 0.093 | 0.101 |  |
| Whole Brain Volume | Mood or Anxiety Disorder (Yes) | -0.009 | -0.043 | 0.025 | 0.017 | -0.016 | 0.602 |  | 0.004 | -0.017 | 0.025 | 0.011 | 0.014 | 0.688 |  |
|  | Parental Education | **0.005** | **0.002** | **0.007** | **0.001** | **0.146** | **<0.001** |  | **0.003** | **0.001** | **0.005** | **0.001** | **0.110** | **0.001** |  |
|  | Recruitment Centre (Dresden) | **-0.048** | **-0.075** | **-0.021** | **0.014** | **-0.151** | **0.001** |  | -0.004 | -0.027 | 0.020 | 0.012 | -0.013 | 0.761 |  |
|  | Recruitment Centre (Dublin) | **0.048** | **0.016** | **0.081** | **0.016** | **0.135** | **0.003** |  | **0.062** | **0.037** | **0.087** | **0.013** | **0.187** | **<0.001** |  |
|  | Recruitment Centre (Hamburg) | **-0.035** | **-0.064** | **-0.007** | **0.014** | **-0.106** | **0.015** |  | -0.014 | -0.036 | 0.009 | 0.012 | -0.049 | 0.245 |  |
|  | Recruitment Centre (London) | 0.022 | -0.007 | 0.050 | 0.015 | 0.064 | 0.137 |  | 0.009 | -0.014 | 0.031 | 0.012 | 0.031 | 0.459 |  |
|  | Recruitment Centre (Mannheim) | **-0.033** | **-0.060** | **-0.006** | **0.014** | **-0.094** | **0.018** |  | -0.022 | -0.045 | 0.001 | 0.012 | -0.074 | 0.060 |  |
|  | Recruitment Centre (Nottingham) | 0.006 | -0.021 | 0.034 | 0.014 | 0.022 | 0.664 |  | 0.016 | -0.006 | 0.038 | 0.011 | 0.062 | 0.164 |  |
|  | Recruitment Centre (Paris) | 0.008 | -0.023 | 0.039 | 0.016 | 0.026 | 0.600 |  | 0.003 | -0.022 | 0.028 | 0.013 | 0.011 | 0.814 |  |
|  | Mean PDS score | **0.016** | **0.004** | **0.028** | **0.006** | **0.078** | **0.011** |  | 0.008 | -0.006 | 0.023 | 0.007 | 0.038 | 0.248 |  |
|  | Family Support | 0.006 | -0.001 | 0.013 | 0.004 | 0.062 | 0.115 |  | 0.003 | -0.005 | 0.010 | 0.004 | 0.030 | 0.502 |  |
|  | Peer Problems | 0.001 | -0.007 | 0.010 | 0.004 | 0.012 | 0.787 |  | -0.003 | -0.011 | 0.005 | 0.004 | -0.035 | 0.446 |  |
|  | Socioeconomic Stress | -0.003 | -0.013 | 0.007 | 0.005 | -0.028 | 0.535 |  | **-0.009** | **-0.018** | **-0.001** | **0.004** | **-0.105** | **0.030** |  |
| Amygdala GMV | Mood or Anxiety Disorder (Yes) | -0.010 | -0.149 | 0.128 | 0.071 | -0.004 | 0.885 |  | -0.069 | -0.147 | 0.009 | 0.040 | -0.050 | 0.083 |  |
|  | Parental Education | -0.003 | -0.012 | 0.006 | 0.004 | -0.023 | 0.506 |  | 0.005 | -0.002 | 0.012 | 0.004 | 0.040 | 0.164 |  |
|  | Recruitment Centre (Dresden) | 0.054 | -0.049 | 0.158 | 0.053 | 0.042 | 0.301 |  | 0.038 | -0.053 | 0.128 | 0.046 | 0.030 | 0.413 |  |
|  | Recruitment Centre (Dublin) | -0.031 | -0.149 | 0.086 | 0.060 | -0.021 | 0.601 |  | -0.092 | -0.184 | 0.000 | 0.047 | -0.062 | 0.051 |  |
|  | Recruitment Centre (Hamburg) | **0.117** | **0.006** | **0.229** | **0.057** | **0.086** | **0.039** |  | 0.006 | -0.082 | 0.095 | 0.045 | 0.005 | 0.888 |  |
|  | Recruitment Centre (London) | **-0.133** | **-0.242** | **-0.025** | **0.056** | **-0.097** | **0.016** |  | **-0.289** | **-0.378** | **-0.201** | **0.045** | **-0.235** | **<0.001** |  |
|  | Recruitment Centre (Mannheim) | 0.050 | -0.044 | 0.145 | 0.048 | 0.035 | 0.296 |  | -0.056 | -0.142 | 0.030 | 0.044 | -0.043 | 0.199 |  |
|  | Recruitment Centre (Nottingham) | **0.209** | **0.118** | **0.300** | **0.046** | **0.184** | **<0.001** |  | **0.181** | **0.102** | **0.261** | **0.041** | **0.162** | **<0.001** |  |
|  | Recruitment Centre (Paris) | 0.051 | -0.055 | 0.157 | 0.054 | 0.039 | 0.348 |  | 0.001 | -0.090 | 0.090 | 0.046 | 0.001 | 0.997 |  |
|  | Mean PDS score | 0.034 | -0.015 | 0.084 | 0.025 | 0.042 | 0.172 |  | 0.035 | -0.020 | 0.089 | 0.028 | 0.036 | 0.214 |  |
|  | Whole Brain Volume | **1.812** | **1.586** | **2.039** | **0.116** | **0.444** | **<0.001** |  | **2.318** | **2.103** | **2.533** | **0.110** | **0.523** | **<0.001** |  |
|  | Family Support | -0.008 | -0.036 | 0.019 | 0.014 | -0.022 | 0.546 |  | 0.002 | -0.024 | 0.028 | 0.013 | 0.005 | 0.882 |  |
|  | Peer Problems | -0.008 | -0.043 | 0.027 | 0.018 | -0.020 | 0.655 |  | 0.023 | -0.005 | 0.052 | 0.015 | 0.062 | 0.112 |  |
|  | Socioeconomic Stress | -0.032 | -0.072 | 0.007 | 0.020 | -0.070 | 0.108 |  | 0.001 | -0.031 | 0.032 | 0.016 | 0.002 | 0.970 |  |
| vmPFC GMV | Mood or Anxiety Disorder (Yes) | -0.009 | -0.324 | 0.306 | 0.161 | -0.001 | 0.955 |  | 0.140 | -0.063 | 0.343 | 0.104 | 0.032 | 0.177 |  |
|  | Parental Education | 0.021 | -0.001 | 0.044 | 0.012 | 0.050 | 0.063 |  | -0.011 | -0.030 | 0.008 | 0.010 | -0.029 | 0.240 |  |
|  | Recruitment Centre (Dresden) | -0.280 | -0.576 | 0.017 | 0.151 | -0.065 | 0.064 |  | -0.090 | -0.344 | 0.164 | 0.130 | -0.023 | 0.487 |  |
|  | Recruitment Centre (Dublin) | **0.341** | **0.034** | **0.648** | **0.157** | **0.070** | **0.029** |  | **0.660** | **0.419** | **0.902** | **0.123** | **0.143** | **<0.001** |  |
|  | Recruitment Centre (Hamburg) | 0.175 | -0.128 | 0.479 | 0.155 | 0.039 | 0.258 |  | -0.020 | -0.254 | 0.214 | 0.119 | -0.005 | 0.868 |  |
|  | Recruitment Centre (London) | 0.047 | -0.244 | 0.338 | 0.149 | 0.010 | 0.752 |  | 0.080 | -0.144 | 0.304 | 0.114 | 0.021 | 0.486 |  |
|  | Recruitment Centre (Mannheim) | **-0.384** | **-0.663** | **-0.106** | **0.142** | **-0.081** | **0.007** |  | **-0.303** | **-0.545** | **-0.061** | **0.123** | **-0.074** | **0.014** |  |
|  | Recruitment Centre (Nottingham) | **0.410** | **0.127** | **0.692** | **0.144** | **0.109** | **0.004** |  | **0.388** | **0.155** | **0.621** | **0.119** | **0.111** | **0.001** |  |
|  | Recruitment Centre (Paris) | -0.075 | -0.414 | 0.263 | 0.173 | -0.018 | 0.663 |  | 0.009 | -0.251 | 0.270 | 0.133 | 0.002 | 0.943 |  |
|  | Mean PDS score | -0.086 | -0.203 | 0.031 | 0.060 | -0.031 | 0.150 |  | -0.060 | -0.189 | 0.068 | 0.066 | -0.020 | 0.356 |  |
|  | Whole Brain Volume | **9.021** | **8.439** | **9.603** | **0.297** | **0.667** | **<0.001** |  | **8.793** | **8.177** | **9.409** | **0.314** | **0.635** | **<0.001** |  |
|  | Family Support | 0.038 | -0.036 | 0.113 | 0.038 | 0.030 | 0.309 |  | 0.001 | -0.076 | 0.079 | 0.039 | 0.001 | 0.974 |  |
|  | Peer Problems | 0.021 | -0.064 | 0.106 | 0.043 | 0.016 | 0.626 |  | -0.039 | -0.116 | 0.038 | 0.039 | -0.033 | 0.316 |  |
|  | Socioeconomic Stress | -0.001 | -0.106 | 0.105 | 0.054 | 0.001 | 0.990 |  | -0.053 | -0.137 | 0.030 | 0.043 | -0.043 | 0.212 |  |
| Covariance | Amygdala and vmPFC | 0.004 | -0.020 | 0.027 | 0.012 | 0.009 | 0.766 |  | -0.008 | -0.026 | 0.009 | 0.009 | -0.028 | 0.343 |  |

Note: Statistically significant values (p < .05) are in bold for ease of reading. For recruitment centre, the reference category is Berlin. WBV values were divided by 1,000,000. Amygdala and vmPFC GMV values were divided by 1,000. Model fit: robust χ^2^ = 1006.579, p-value < 0.001, robust CFI = 0.935, robust RMSEA = 0.024 [0.021, 0.026].
