# Supplementary material for: Social environment and brain structure in adolescent mental health: A cross-sectional structural equation modelling study using IMAGEN data
Source: PLoS One. 2023 Jan 5;18(1):e0280062. doi: 10.1371/journal.pone.0280062 (PMC9815590; doi:10.1371/journal.pone.0280062)
Supplement: Attachment — Submitted filename: Response to Reviewers.docx [file pone.0280062.s002.docx]

Response to Reviewers

10^th^ October 2022

Dear Academic Editor,

Thank you for your thoughtful comments for the submission to *PLOS ONE* entitled “Social environment and brain structure in adolescent mental health: A cross-sectional structural equation modelling study using IMAGEN data”.

Please find our response to your additional comments below.

Journal Requirements:

| Comment | Response |
| --- | --- |
| Please review your reference list to ensure that it is complete and correct. If you have cited papers that have been retracted, please include the rationale for doing so in the manuscript text, or remove these references and replace them with relevant current references. Any changes to the reference list should be mentioned in the rebuttal letter that accompanies your revised manuscript. If you need to cite a retracted article, indicate the article’s retracted status in the References list and also include a citation and full reference for the retraction notice. | We have reviewed the reference list and we believe that it is complete and correct. We have not found any papers that have been retracted. If you are aware of retracted papers, please let us know and we will address this. |

Additional Editor Comments:

| Comment | Response |
| --- | --- |
| 1. Double check grammar again | We have made minor grammatical changes throughout the manuscript, which can be seen in the Tracked Changes version. |
| 2. Make sure that all necessary files are on OSF | We cannot upload the IMAGEN data to OSF as they are third party data, as explained in the “Data availability” section of the submission.  We have uploaded the R analysis script to OSF: <https://osf.io/qwaz3> |
| 3. Avoid the excessive lengthy paragraphs | We have restructured some paragraphs and added in information about assumptions checks as requested in point 4. This has helped break up longer paragraphs. |
| 4. Report all stats parameters (assumptions check, effect sizes and CIs) | We have explicitly added in a section about the assumptions checks in the “Analytic strategy” section. The unstandardised (estimate) and standardised (beta) coefficients have already been reported in the Supplementary Tables, which are the effect sizes in regression. We have added confidence intervals to all tables containing regression output – please see the Supplementary Tables. |
| 5. Provide a significance statement at the very end of the discussion. Don’t worry, it can be simple: limitations WITH further recommendations for researchers or next studies & the strengths of your findings. A smoother conclusion is also interesting | We have included a “Significance statement” section at the end of the discussion. |

Sincerely,

Jessica Stepanous
PhD Student, Division of Neuroscience and Experimental Psychology
Zochonis Building, University of Manchester, Manchester, UK
